# Silencing of SIRPα enhances the antitumor efficacy of CAR-M in solid tumors

**DOI:** 10.1038/s41423-024-01220-3

**Published:** 2024-10-08

**Authors:** Han Zhang, Yi Huo, Wenjing Zheng, Peng Li, Hui Li, Lingling Zhang, Longqi Sa, Yang He, Zihao Zhao, Changhong Shi, Lequn Shan, Angang Yang, Tao Wang

**Affiliations:** 1 State Key Laboratory of Holistic Integrative Management of Gastrointestinal Cancers, Department of Medical Genetics and Developmental Biology, Fourth Military Medical University, Xi’an, China; 2 Department of Spine Surgery, Honghui Hospital, Xi’an Jiaotong University, Xi’an, China; 3 State Key Laboratory of Holistic Integrative Management of Gastrointestinal Cancers, Department of Immunology, Fourth Military Medical University, Xi’an, China; 4 Division of Cancer Biology, Laboratory Animal Center, Fourth Military Medical University, Xi’an, China

**Keywords:** Cancer immunotherapy, CAR-M, SIRPα, Phagocytosis, Solid tumor, Cancer immunotherapy, Cancer immunotherapy

## Abstract

The potential of macrophage-mediated phagocytosis as a cancer treatment is promising. Blocking the CD47–SIRPα interaction with a CD47-specific antibody significantly enhances macrophage phagocytosis. However, concerns regarding their toxicity to nontumor cells remain substantial. Here, we engineered chimeric antigen receptor macrophages (CAR-Ms) by fusing a humanized single-chain variable fragment with FcγRIIa and integrating short hairpin RNA to silence SIRPα, thereby disrupting the CD47–SIRPα signaling pathway. These modified CAR-shSIRPα-M cells exhibited an M1-like phenotype, superior phagocytic function, substantial cytotoxic effects on HER2-positive tumor cells, and the ability to eliminate patient-derived organoids. In vivo, CAR-M cells significantly inhibited tumor growth and prolonged survival in tumor-bearing mice. Notably, CAR-shSIRPα-M cells enhanced cytotoxic T-cell infiltration into tumors, thereby enhancing the antitumor response in both the humanized immune system mouse model and immunocompetent mice. Mechanistically, SIRPα inhibition activated inflammatory pathways and the cGAS-STING signaling cascade in CAR-M cells, leading to increased production of proinflammatory cytokines, reactive oxygen species, and nitric oxide, thereby enhancing their antitumor effects. These findings underscore the potential of SIRPα inhibition as a novel strategy to increase the antitumor efficacy of CAR-M cells in cancer immunotherapy, particularly against solid tumors.

## Introduction

Adoptive cell therapy, particularly chimeric antigen receptor T-cell (CAR-T) therapy, has made remarkable progress in the treatment of hematological malignancies. However, its efficacy against solid tumors has not met expectations, primarily because of challenges related to tumor infiltration and the suppressive nature of the immune microenvironment [1–3]. An increase in the therapeutic potency of CAR-T cells in solid tumors necessitates the development of strategies that refine CAR design or employ combination immunotherapy [2, 4]. Alternatively, applying CARs to other immune cells may present a novel approach for cancer immunotherapy [4, 5].

Macrophages exhibit a strong capacity for tumor infiltration and constitute the predominant immune cell population in most solid tumors [6]. They can directly eliminate tumor cells through phagocytosis and attract other immune cells by releasing cytokines and chemokines. Furthermore, macrophages can activate T cells and induce adaptive immune responses via antigen presentation [6, 7]. This multifaceted functionality makes macrophages ideal vectors for CAR constructs. In a landmark study in 2020, Klichinsky et al. provided pioneering evidence for CAR-M cells [8]. By using the adenovirus vector Ad5f35, they engineered macrophages that demonstrated proficient phagocytosis and destruction of malignant cells both in vitro and in vivo. Since then, CAR-M research has garnered increasing attention and has become a major focal point in the field of cancer immunotherapy [9–16].

CARs are synthetic transmembrane receptors that enable immune cells to target and eliminate cells expressing specific ligands. Generally, CARs consist of an extracellular single-chain variable fragment (scFv), a transmembrane domain, and an intracellular signaling domain. The activation of the intracellular domain occurs upon scFv-mediated target recognition, triggering downstream signaling. The choice of the intracellular signaling domain for CAR-M cells varies among research groups. Some retain the T-cell CD3ζ activation module to maintain macrophage activation [8, 12, 15, 17], whereas others incorporate modules from toll-like receptors (TLRs) or Fc receptors (FcRs) as intracellular signaling domains [9, 10, 16, 18, 19]. Although CAR-M cells with the CD3ζ module can identify and eliminate tumor cells, the mechanism differs from that of CAR-T cells. Therefore, the extent to which CD3ζ, which is absent in macrophages, can stimulate the antitumor activity of macrophages warrants further research. TLRs, which are essential in the innate immune system for recognizing pathogen-associated molecular patterns (PAMPs), have proven effective as intracellular domains in CAR-M cells for clearing tumor cells. These domains activate inflammatory signaling pathways under antigenic stimulation [9, 18]. Similarly, the FcR module can also serve as an intracellular signal transduction domain for CAR-M [10, 16, 19]. FcRs, which bind the Fc region of immunoglobulins, play crucial roles in mediating antibody functions such as antibody-dependent cellular cytotoxicity, antibody-dependent cellular phagocytosis (ADCP), and cytokine and chemokine stimulation [20, 21]. Considering the ability of FcγRs to facilitate various antitumor effects, their incorporation into CARs could enhance the antitumor activity of macrophages.

Phagocytosis is a vital mechanism by which macrophages eliminate tumor cells and activate the immune system. This process is regulated by receptor‒ligand interactions, which signal that macrophages either promote or inhibit phagocytosis [22, 23]. The CD47-SIRPα signaling pathway serves as a key phagocytic checkpoint, with tumor cells often overexpressing CD47 to evade phagocytosis [24, 25]. Blocking CD47 with monoclonal antibodies or soluble SIRPα-Fc can trigger macrophage-mediated ADCP and significantly kill tumor cells [26–29]. Additionally, inhibiting CD47-SIRPα can activate T cells via macrophage antigen presentation, stimulating adaptive antitumor immune responses [30]. CD47-targeted antibodies also have synergistic effects with other antitumor monoclonal antibodies, increasing their efficacy [31, 32]. However, the widespread expression of CD47 in nontumor cells poses challenges, as targeting CD47 can lead to blood toxicity, including agglutination and anemia, hindering the clinical advancement of CD47 monoclonal antibodies [33, 34].

In this study, we designed CAR-Ms to target HER2-positive tumors through the engineering of a fusion protein that combines the humanized scFv P1h3 with the Fc receptor FcγRIIa. Additionally, we incorporated a targeted short hairpin RNA (shRNA) cassette to inhibit SIRPα, disrupting the CD47-SIRPα signaling axis. This strategic combination of the “eat me” signal from FcγRIIa and the suppression of the “don’t eat me” signal via SIRPα inhibition endowed CAR-shSIRPα-M with increased phagocytic activity and induced an M1-like phenotype, resulting in significant cytotoxic effects on HER2-positive tumor cells. These modified macrophages successfully eradicated patient-derived organoids (PDOs), substantially reduced tumor growth, and extended the lifespan of tumor-bearing mice. Importantly, blockade of SIRPα facilitated cytotoxic T-cell infiltration into tumors, markedly amplifying the antitumor response. Our findings also suggest that SIRPα inhibition activates inflammatory pathways and cGAS-STING signaling in CAR-Ms, leading to increased production of proinflammatory cytokines. Furthermore, the antitumor properties of CAR-Ms with suppressed SIRPα were enhanced by the generation of reactive oxygen species (ROS) and nitric oxide (NO), both of which are potent oxidizers. Overall, our research introduces a novel and effective strategy for developing CAR-Ms with enhanced antitumor efficacy and proposes a potent cellular immunotherapy approach for the treatment of solid tumors.

## Results

### Chimeric antigen receptors containing FcγRIIa significantly increase targeted phagocytosis in macrophages

**Fig. 1:**
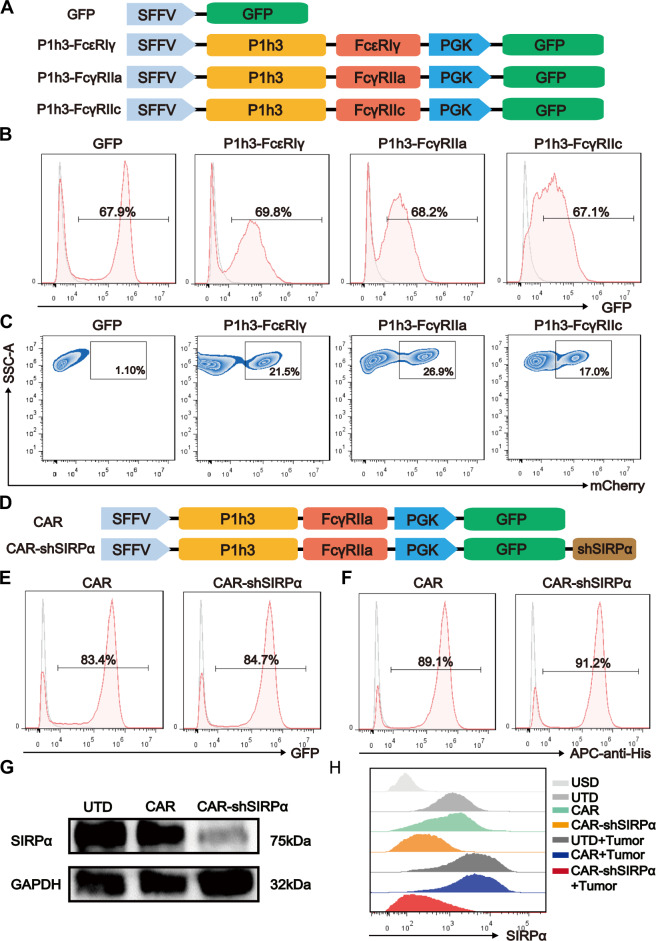
The FcγRIIa domain enhances CAR-mediated tumor phagocytosis. **A** Construction of HER2-targeting CAR macrophages incorporating diverse FcγR domains. **B** Flow cytometry histograms illustrating the lentivirus transfection efficiency in THP-1 cells. **C** Fluorescence-activated cell sorting (FACS) analysis of the phagocytic efficiency of the GFP^+^ macrophages. GFP- and CAR-modified macrophages were cocultured with mCherry^+^ SKOV3 cells at an effector-to-target ratio (E:T) of 1:1 for 1 h before flow cytometry analysis. **D** Structural diagram of CAR and CAR-shSIRPα. Flow cytometry histograms depicting CAR and CAR-shSIRPα expression levels, with GFP (**E**) and His-tagged recombinant HER2 (**F**) in sorted THP-1 cells. **G** Immunoblot analysis of SIRPα protein levels in untransduced (UTD), CAR, and CAR-shSIRPα macrophages. **H** FACS analysis of SIRPα expression levels in unstained (USD), untreated (UTD), CAR, and CAR-shSIRPα macrophages, as well as UTD, CAR, and CAR-shSIRPα macrophages cocultured with SKOV3 cells for 24 h

FcγRs are the primary receptors responsible for engulfing particles bound by antibodies, with the immune receptor tyrosine activation motif (ITAM) playing a key role in this process. To increase the phagocytic function of CARs, we developed receptors by fusing the scFv targeting HER2 (P1h3) with the transmembrane and intracellular domains of FcγRI (FcεRIγ), FcγRIIa, and FcγRIIc, which all contain ITAMs. These recombinant CAR sequences were inserted downstream of the SFFV promoter in a third-generation lentiviral vector, while GFP was cloned under the PGK promoter (Fig. 1A).

To generate CAR-modified macrophages, we first optimized the differentiation conditions for THP-1 human leukemic monocytes. Mature macrophages induced with 20 ng/mL phorbol-12-myristate-13-acetate (PMA) expressed the conventional macrophage markers CD11b and CD14 and maintained high viability (Supplementary Fig. 1a, b). Compared with untransduced (UTD) macrophages, lentiviral infection achieved approximately 65% efficiency in these macrophages (Fig. 1B), with no significant differences observed in terms of proliferation, cell cycle, or viability, regardless of PMA induction (Supplementary Fig. 1c–i). To evaluate the phagocytic capacity of CAR-modified macrophages, we cocultured CAR-Ms with HER2-positive SKOV3 cells that consistently expressed mCherry. Flow cytometry analysis revealed minimal phagocytosis by control GFP macrophages, whereas P1h3-FcεRIγ, P1h3-FcγRIIa, and P1h3-FcγRIIc macrophages efficiently engulfed tumor cells. Notably, P1h3-FcγRIIa macrophages presented the highest phagocytic ratio, suggesting that the presence of FcγRIIa in the CAR structure enhances targeted phagocytosis the most (Fig. 1C). Therefore, we utilized P1h3-FcγRIIa as the chimeric receptor in subsequent experiments, referred to as “CAR.” In addition to the use of the lentiviral system for CAR delivery, we also investigated a replication-incompetent adenoviral vector (Ad5f35), guided by Klichinsky’s study [8]. Our findings showed that macrophage infection efficiency was dependent on the adenoviral dose; however, cell viability decreased significantly at higher multiplicities of infection (MOIs) (Supplementary Fig. 2b–d). We ultimately opted for the lentiviral system in subsequent experiments because of its acceptable infection efficiency, cell viability, and cost-effectiveness.

To further enhance the phagocytic activity of CAR-modified macrophages against tumors, we incorporated a shRNA expression element targeting SIRPα downstream of the GFP sequence in the lentiviral vector and designated it CAR-shSIRPα (Fig. 1D). Postsorting, the proportion of GFP-positive CAR and CAR-shSIRPα macrophages exceeded 83% (Fig. 1E). Moreover, fluorescence-activated cell sorting (FACS) analysis revealed that approximately 90% of the sorted CAR and CAR-shSIRPα macrophages expressed CAR molecules, which was correlated with the presence of GFP expression levels in the CAR (Fig. 1F). Western blot analysis revealed a significant reduction in SIRPα expression in CAR-shSIRPα macrophages compared with that in UTD and CAR macrophages (Fig. 1G and Supplementary Fig. 2e). Importantly, CD47 expression was extremely high in SKOV3, SKBR3, DLD-1, and ASPC1 tumor cells (Supplementary Fig. 2a), and coculture with these tumor cells significantly increased SIRPα expression in UTD and CAR macrophages, whereas CAR-shSIRPα macrophages maintained low SIRPα levels (Fig. 1H). These findings indicate effective inhibition of CD47-SIRPα signaling by CAR-shSIRPα macrophages.

### Inhibition of SIRPα enhances the antitumoral M1-like polarizing phenotype of CAR-modified macrophages

**Fig. 2:**
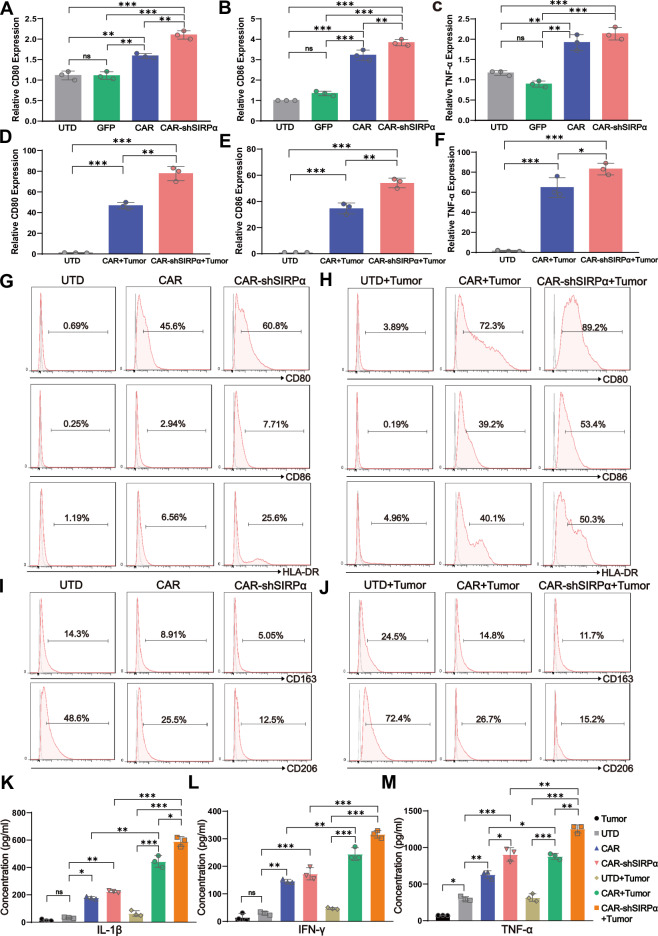
CAR-shSIRPα-treated macrophages exhibit an enhanced M1-like polarization phenotype. qRT‒PCR analysis of CD80 (**A**), CD86 (**B**), and TNF-α (**C**) expression levels in UTD, GFP, CAR, and CAR-shSIRPα macrophages. qRT‒PCR analysis of CD80 (**D**), CD86 (**E**), and TNF-α (**F**) expression levels in UTD-stimulated, and SKOV3-cocultured CAR- and CAR-shSIRPα-stimulated macrophages. **G**–**J** FACS analysis of M1- and M2-like phenotypic markers in resting macrophages and macrophages cocultured with SKOV3 cells. Expression levels of CD80, CD86, and HLA-DR in resting (**G**) and SKOV3-cocultured (**H**) UTD, CAR, and CAR-shSIRPα macrophages. Expression of CD163 and CD206 in resting (**I**) and SKOV3-cocultured (**J**) UTD, CAR, and CAR-shSIRPα macrophages. ELISA analysis of IL-1β (**K**), INF-γ (**L**), and TNF-α (**M**) production in UTD, CAR, and CAR-shSIRPα macrophages with or without coculture with SKOV3 cells. The data are presented as the means ± s.e.m. of three technical replicates. For all panels, *P < 0.05, **P < 0.01, ***P < 0.001

Macrophages exhibit significant phenotypic and functional diversity. While M1-like macrophages possess antitumor properties, most tumor-associated macrophages (TAMs) undergo polarization toward a tumor-promoting M2-like phenotype in response to signals from the tumor microenvironment (TME), contributing to rapid tumor progression. Therefore, maintaining an antitumoral phenotype in CAR-modified macrophages following incubation with tumor cells is essential to ensure sustained antitumor activity. To this end, we performed phenotypic characterization of CAR macrophages and CAR-shSIRPα macrophages with and without antigen stimulation. Our qPCR and flow cytometry results indicated that CAR macrophages and CAR-shSIRPα macrophages presented significantly higher expression levels of CD80, CD86, and TNF-α than control macrophages (UTD and GFP) in the resting state. The macrophages in which SIRPα was knocked down presented the highest levels of CD80, CD86 and TNF-α expression (Fig. 2A–C). Coculture with SKOV3 cells led to a notable increase in M1-like polarization markers in CAR-modified macrophages. Consistent with the resting-state phenotype, CAR-shSIRPα macrophages presented higher expression levels of CD80, CD86, and TNF-α than CAR-Ms did (Fig. 2D–F). The upregulation of CD80, CD86, and HLA-DR in CAR-modified macrophages was further confirmed by FACS analysis (Fig. 2G, H). Although coculture with SKOV3 cells increased the expression of the M2-like phenotype markers CD163 and CD206, CAR-M and CAR-shSIRPα-M presented significantly lower expression levels of CD163 and CD206 than did UTD macrophages. The lowest expression levels were observed in CAR-shSIRPα-M under both resting and antigen-stimulated conditions (Fig. 2I, J). Furthermore, CAR-M and CAR-shSIRPα-M exhibited increased secretion of IL-1β, IFN-γ, and TNF-α, regardless of tumor antigen stimulation. Notably, CAR-shSIRPα-M presented the highest capacity for proinflammatory cytokine secretion (Fig. 2K–M). These results illustrate that CAR-M and CAR-shSIRPα-M display an antitumor M1-like polarization phenotype and that SIRPα inhibition further enhances the ability of CAR-modified macrophages to present antigens and secrete proinflammatory cytokines.

### Silencing of SIRPα in CAR-modified macrophages results in significant antigen-specific phagocytosis and cytotoxicity against HER2-positive tumor cells in vitro

**Fig. 3:**
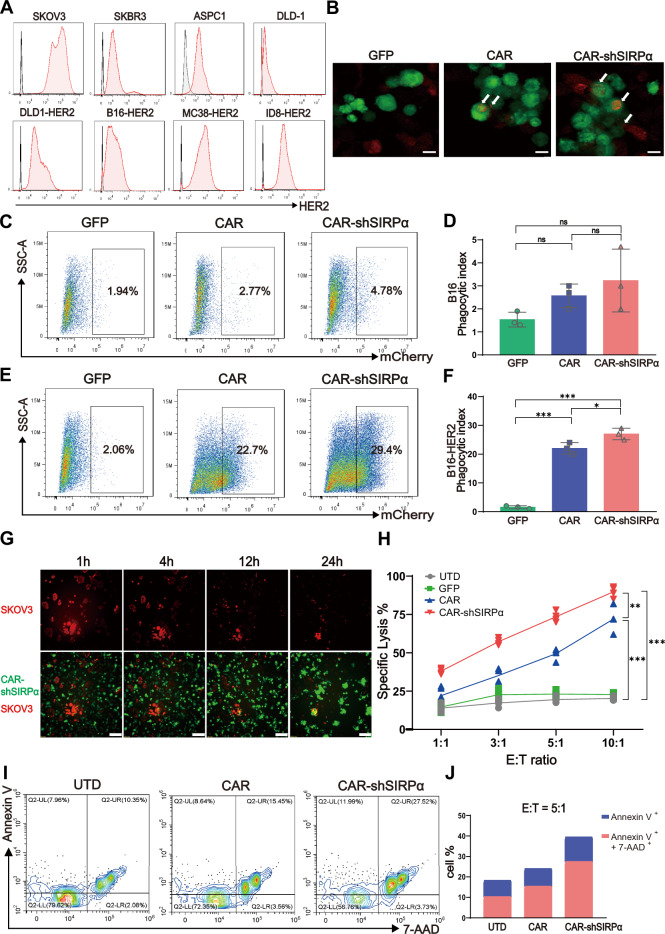
CAR-shSIRPα macrophages exhibit enhanced tumor phagocytosis and cytotoxicity. **A** FACS analysis of HER2 expression levels in human and mouse tumor cell lines. **B** Confocal microscopy analysis of macrophage phagocytosis. mCherry^+^ SKOV3 cells were cocultured with GFP, CAR, or CAR-shSIRPα macrophages at an effector-to-target (E:T) ratio of 5:1 for 1 h before visualization. Scale bars represent 20 μm. **C**–**F** FACS analysis of targeted tumor phagocytosis by GFP^+^ macrophages. HER2-negative B16 cells (**C**) and HER2-positive B16-HER2 cells (**E**) were cocultured with GFP, CAR, or CAR-shSIRPα macrophages at an E:T ratio of 1:1 for 1 h prior to analysis. Statistical analysis of the phagocytosis efficiency of HER2-negative B16 cells (**D**) and HER2-positive B16-HER2 cells (**F**). **G** Cytotoxic effects of CAR-modified macrophages against HER2-positive tumor cells. mCherry-positive SKOV3 cells were cocultured with GFP, CAR, or CAR-shSIRPα macrophages at an E:T ratio of 5:1 for 24 h. Continuous images were obtained via a Lumascope 720 fluorescence microscope. Scale bars represent 100 μm. **H** Luciferase-based cytotoxicity analysis of CAR-modified macrophages at E:T ratios of 1:1, 3:1, 5:1, and 10:1 against SKOV3 cells after 24 h of coculture. **I** Apoptosis analysis of CAR-modified macrophages cocultured with SKOV3 cells. **J** Statistical analysis of the results presented in (**I**). The data are presented as the means ± s.e.m. of three technical replicates. For all panels, *P < 0.05, **P < 0.01, ***P < 0.001

To explore the specific phagocytic effect of CAR-modified macrophages on HER2-positive tumor cells, we assessed HER2 antigen expression in various tumor cell lines. High HER2-positive rates were observed in the human SKOV3, SKBR3, and ASPC1 cancer cell lines, with SKOV3 cells demonstrating the highest HER2 antigen expression level. Conversely, human DLD-1 cancer cells presented low HER2 abundance (Fig. 3A). Subsequently, DLD-1 cells and mouse B16, MC-38, and ID8 cancer cells were transduced with lentiviruses carrying truncated human HER2 lacking intracellular domains (Fig. 3A). The externally introduced truncated HER2 antigens enabled CAR-modified macrophages to selectively target tumor cells while avoiding interference from HER2 signaling. Furthermore, lentiviral transduction enabled the stable expression of mCherry and luciferase in these cell lines, facilitating both in vitro and in vivo experiments.

Using confocal microscopy, we observed clear antigen-specific phagocytosis of mCherry-positive SKOV3 tumor cells by CAR-modified macrophages (Fig. 3B). Furthermore, B16 and B16-HER2 cells were cocultured with CAR-modified macrophages, and subsequent phagocytosis was quantitatively assessed via flow cytometry to evaluate the specific engulfment of tumor cells by CAR-modified macrophages. The results revealed that GFP control macrophages, as well as CAR-M and CAR-shSIRPα-M, exhibited low levels of phagocytosis of HER2-negative B16 cells (Fig. 3C, D). In contrast, while control macrophages maintained low phagocytosis rates, CAR-M and CAR-shSIRPα-M demonstrated phagocytosis rates of 22.7% and 29.4%, respectively, for HER2-positive B16-HER2 cells (Fig. 3E, F), suggesting that the phagocytic activity of CAR-modified macrophages toward tumors is HER2 specific. Additionally, we conducted a comparative analysis of targeted phagocytosis by CAR-modified macrophages in HER2-positive SKOV3, SKBR3, ID8-HER2, MC38-HER2, and DLD1-HER2 cell lines. Our results illustrate that CAR molecules equip macrophages with effective targeted phagocytic capabilities and that further suppression of SIRPα can enhance this phagocytic effect (Supplementary Fig. 3).

To determine whether knocking down SIRPα universally enhances the phagocytic ability of CAR-modified macrophages, we replaced P1h3 with FMC63, a scFv against human CD19, and constructed CD19-targeting CAR-Ms and CAR-shSIRPα-Ms (Supplementary Fig. 4a). Through coculture phagocytosis experiments, we observed that CAR-shSIRPα-Ms exhibited significantly greater phagocytic activity against CD19-positive human Burkitt lymphoma Raji cells and human diffuse large B-cell lymphoma SU-DHL-4 cells than CAR-Ms did (Supplementary Fig. 4b). These findings suggest that SIRPα knockdown enhances the phagocytic ability of CAR-modified macrophages that target various antigens.

Next, we investigated the cytotoxicity of CAR-modified macrophages to HER2-positive tumor cells. Our results showed that within 24 h of coculture, CAR-modified macrophages effectively eliminated SKOV3 cells (Fig. 3G). Furthermore, cytotoxicity experiments at various effector-to-target (E:T) ratios demonstrated that both CAR and CAR-shSIRPα macrophages had significant dose-dependent killing effects on HER2-positive tumor cells (Fig. 3H and Supplementary Fig. 5a–d). Additionally, coculture with CAR-modified macrophages resulted in notable induction of apoptosis in tumor cells, with CAR-shSIRPα demonstrating an even more pronounced proapoptotic effect (Fig. 3I, J and Supplementary Fig. 5e–j). Overall, these findings suggest that CAR-modified macrophages exhibit promising antitumor effects against HER2-positive tumor cells in vitro.

### CAR-modified macrophages exhibit potent antitumor activity in patient-derived organoids

**Fig. 4:**
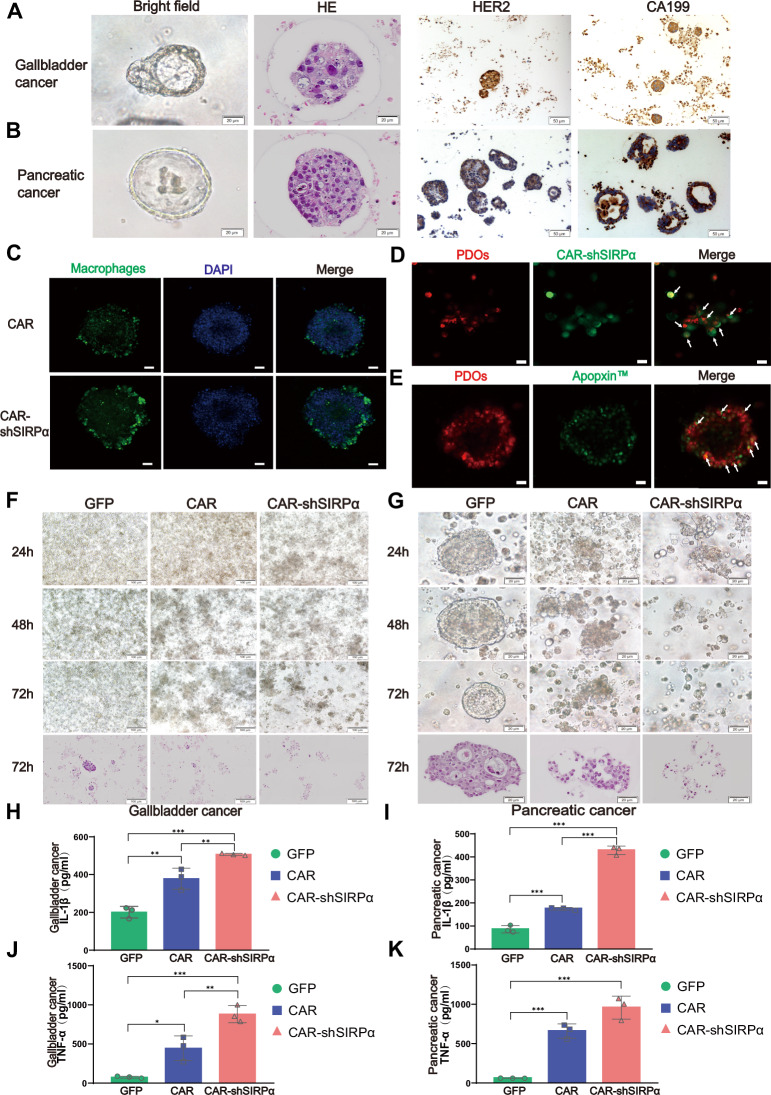
CAR-modified macrophages effectively eradicate tumor patient-derived organoids (PDOs). Cultivation of gallbladder cancer PDOs (**A**) and pancreatic cancer PDOs (**B**). The morphology of the PDOs was examined via bright-field microscopy. Additionally, hematoxylin and eosin (HE) staining and immunohistochemical (IHC) staining were performed to assess the expression of HER2 and CA199 in the PDOs. Scale bars represent 20 μm for bright-field and HE-stained images and 50 μm for IHC-stained images. **C** Confocal imaging of the accumulation of CAR-modified macrophages around PDOs. PDOs were cocultured with CAR- and CAR-shSIRPα-treated macrophages (green) for 12 h. Nuclei were stained prior to confocal imaging. Scale bars represent 20 μm. **D** Phagocytosis of PDOs by CAR-shSIRPα macrophages. PDOs were stained with CellTracker (red) and cocultured with CAR-shSIRPα macrophages (green) for 48 h before confocal imaging. Scale bars represent 50 μm. **E** Cytotoxic effects of CAR-shSIRPα macrophages on PDO cells. PDOs labeled with CellTracker (red) were cocultured with CAR-shSIRPα macrophages that did not express GFP for 48 h, after which Apopxin™ Green was added to identify apoptotic cells before confocal imaging. Scale bars represent 50 μm. Destruction of the PDO structure by CAR-modified macrophages. Gallbladder cancer PDOs (**F**, scale bars represent 100 μm) or pancreatic cancer PDOs (**G**, scale bars represent 20 μm) were cocultured with GFP, CAR, and CAR-shSIRPα macrophages. Bright-field microscopy and HE were performed at the specified time points. **H**–**K** Identification of proinflammatory cytokines in the supernatants of the cocultures. Gallbladder cancer PDOs (**H**, **J**) and pancreatic cancer PDOs (**I**, **K**) were cocultured with GFP, CAR, and CAR-shSIRPα macrophages for 72 h. The supernatant was analyzed via ELISA to measure the levels of IL-1β (**H**, **I**) and TNF-α (**J**, **K**). For all panels, *P < 0.05, **P < 0.01, ***P < 0.001

Compared with traditional tumor cell lines, PDOs provide a more accurate representation of tumor phenotypes and characteristics. To validate the targeted killing capability of CAR-modified macrophages, primary tumors from gallbladder and pancreatic cancer patients were isolated, and stable, expandable organoids were subsequently generated. Bright-field observations and hematoxylin and eosin (HE) staining revealed the characteristic features of the spherical organoids. Immunohistochemistry confirmed the concurrent expression of HER2 and CA199 tumor antigens in the constructed PDOs (Fig. 4A, B, and Supplementary Fig. 6a). Coculturing macrophages with PDOs revealed that CAR-modified macrophages adhered to the PDOs and efficiently engulfed the tumor cells (Fig. 4C, D, Supplementary Fig. 6b–e). After 48 h of coculture, Apopxin™ Green was added to detect apoptosis, revealing that PDO tumor cells died when cocultured with CAR-shSIRPα macrophages (Fig. 4E). Bright-field observations and HE staining revealed considerable disruption of the PDO structure within 72 h, particularly in CAR- and CAR-shSIRPα-treated macrophages. Notably, CAR-shSIRPα macrophages had a more significant damaging effect on HER2-positive PDOs (Fig. 4F, G). ELISAs revealed elevated levels of IL-1β and TNF-α in the coculture medium of PDOs and CAR-modified macrophages, with the highest concentrations recorded in the CAR-shSIRPα macrophages (Fig. 4H–K).

### Inhibition of SIRPα enhances inflammatory signaling, triggers the cGAS-STING pathway, and augments the production of ROS and NO

**Fig. 5:**
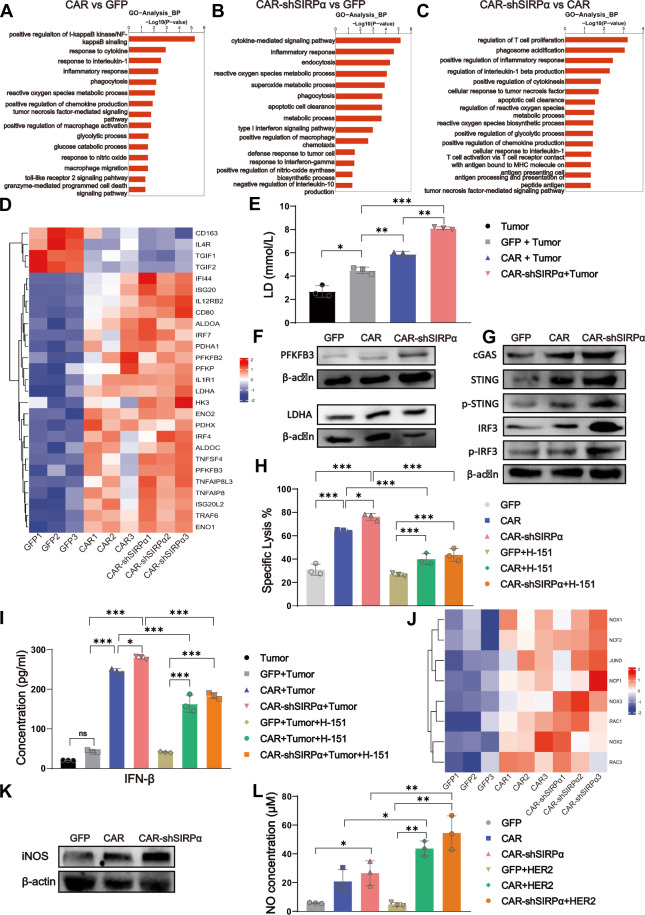
Enhanced inflammatory signaling, cGAS-STING pathway activation, and ROS and NO production in CAR-shSIRPα macrophages. SKOV3 cells cocultured with GFP, CAR, or CAR-shSIRPα macrophages were sorted via flow cytometry and subsequently subjected to RNA sequencing. Gene Ontology (GO) term enrichment analysis of differentially expressed genes (DEGs) was conducted for CAR *vs*. GFP (**A**), CAR-shSIRPα *vs*. GFP (**B**), and CAR-shSIRPα *vs*. CAR (**C**). **D** Heatmap analysis of DEGs related to polarization and glycolysis in GFP, CAR, and CAR-shSIRPα macrophages. **E** Lactate levels were detected in the supernatants of GFP, CAR, and CAR-shSIRPα macrophages cocultured with SKOV3 cells for 24 h. Immunoblot analysis of PFKFB3 and LDHA (**F**) and cGAS-STING signaling (**G**) protein levels in GFP, CAR, and CAR-shSIRPα-sorted macrophages stimulated with SKOV3 cells at an E:T ratio of 10:1 for 24 h. **H** Luciferase-based cytotoxicity analysis of CAR-modified macrophages at E:T ratios of 10:1 against SKOV3 cells after 24 h of coculture, with or without the STING inhibitor H-151. (**I**) ELISA analysis of INF-β production in CAR-modified macrophages at E:T ratios of 10:1 against SKOV3 cells after 24 h of coculture with or without the STING inhibitor H-151. **J** Heatmap analysis of DEGs related to NADPH oxidase complex-related genes in GFP, CAR, and CAR-shSIRPα macrophages. **K** Immunoblot analysis of iNOS protein levels in GFP-, CAR-, and CAR-shSIRPα-sorted macrophages stimulated with SKOV3 cells. **L** NO production by GFP, CAR, and CAR-shSIRPα macrophages with or without recombinant HER2 stimulation. *P < 0.05, **P < 0.01

To elucidate the potential mechanisms underlying the targeted engulfment and killing of tumor cells by CAR-modified macrophages, we analyzed the expression profiles of control, CAR, and CAR-shSIRPα macrophages. Transcriptomic sequencing data were subjected to Gene Ontology (GO) analysis, which revealed that, compared with those in the UTD control group, CAR macrophages presented enrichment of genes associated with biological processes such as the regulation of NF-κB signaling, the response to IL-1, the inflammatory response, and phagocytosis (Fig. 5A). Conversely, CAR-shSIRPα-M presented significant enrichment of genes related to biological processes such as cytokine-mediated signaling pathways, the inflammatory response, endocytosis, ROS metabolic processes, and superoxide metabolic processes (Fig. 5B). Furthermore, compared with CAR macrophages, CAR-shSIRPα macrophages not only presented further enrichment of genes linked to inflammatory responses, phagocytosis, and antigen presentation but also presented additional enrichment of genes associated with processes such as the regulation of T-cell proliferation, the regulation of ROS metabolism, and the regulation of glycolysis (Fig. 5C). Enrichment analysis via the Kyoto Encyclopedia of Genes and Genomes (KEGG) and gene set enrichment analysis (GSEA) revealed that, compared with control macrophages, CAR-modified macrophages were predominantly enriched in inflammatory signaling pathways, including the TNF-α, NF-κB, MAPK, PI3K-Akt, and TLR signaling pathways (Supplementary Fig. 7a–d). In contrast, compared with CAR-shSIRPα macrophages, CAR-shSIRPα macrophages presented enriched signaling pathways related to cytokines, chemokines, phagocytosis, and TLR signaling (Supplementary Fig. 7e, f).

In particular, both CAR- and CAR-shSIRPα-treated macrophages presented increased expression of M1 proinflammatory genes and significantly elevated expression of genes associated with glycolysis, which was particularly pronounced in CAR-shSIRPα-treated macrophages (Fig. 5D). Given the crucial role of glycolysis in M1 macrophage polarization, lactate secretion was quantified in each group. The results indicated that CAR-modified macrophages presented higher levels of lactate expression than control macrophages did, while CAR-shSIRPα macrophages presented the highest lactate expression levels (Fig. 5E). Moreover, Western blot analysis revealed that coincubation with recombinant HER2 protein resulted in a noticeable increase in the expression of key glycolytic enzymes, namely, PFKBR3 and LDHA, in both CAR-M and CAR-shSIRPα-M (Fig. 5F). This observation implies that CAR-modified macrophages may enhance the M1 phenotype through enhanced glycolysis.

Tumor-derived DNA is capable of escaping from phagolysosomes into the cytosol, where it is recognized by cGAS, thereby activating the cGAS-STING signaling pathway. Consistent with these findings, our study revealed significant upregulation of the expression of cGAS, STING, phosphorylated STING, IRF3, and phosphorylated IRF3—key transcription factors within this cascade—in CAR-modified macrophages following coincubation with tumor cells, particularly in SIRPα-inhibited CAR-Ms (Fig. 5G). Furthermore, research indicated that the specific STING inhibitor H-151 substantially reduced the cytotoxicity of CAR-modified macrophages against tumor cells (Fig. 5H) and diminished the secretion of IFN-β by both CAR-Ms and CAR-shSIRPα-Ms upon coincubation with tumor cells (Fig. 5I). These findings highlight the significant role of the cGAS-STING signaling pathway in the antitumor response mediated by CAR-modified macrophages.

Analysis of transcription in CAR-modified macrophages revealed significant enrichment of the ROS and superoxide metabolism signaling pathways. To investigate the contributions of ROS and superoxide to the antitumor effect of CAR-modified macrophages, we examined the expression levels of genes related to the NADPH oxidase complex. Our results indicated notable upregulation of genes such as NOX1, NOX2, NOX3, NCF1, NCF2, RAC1, and RAC2 in CAR-modified macrophages, particularly in CAR-shSIRPα-Ms (Fig. 5J). Furthermore, both the expression of inducible nitric oxide synthase (iNOS) and the production of nitric oxide (NO) were significantly increased in CAR-modified macrophages after coincubation with tumors (Fig. 5K, L). This increase in ROS and NO production offers a plausible explanation for the phagocytosis-independent cytotoxic activity observed in CAR-modified macrophages (Fig. 3I, J).

### CAR-shSIRPα macrophages demonstrate significant in vivo antitumor activity

**Fig. 6:**
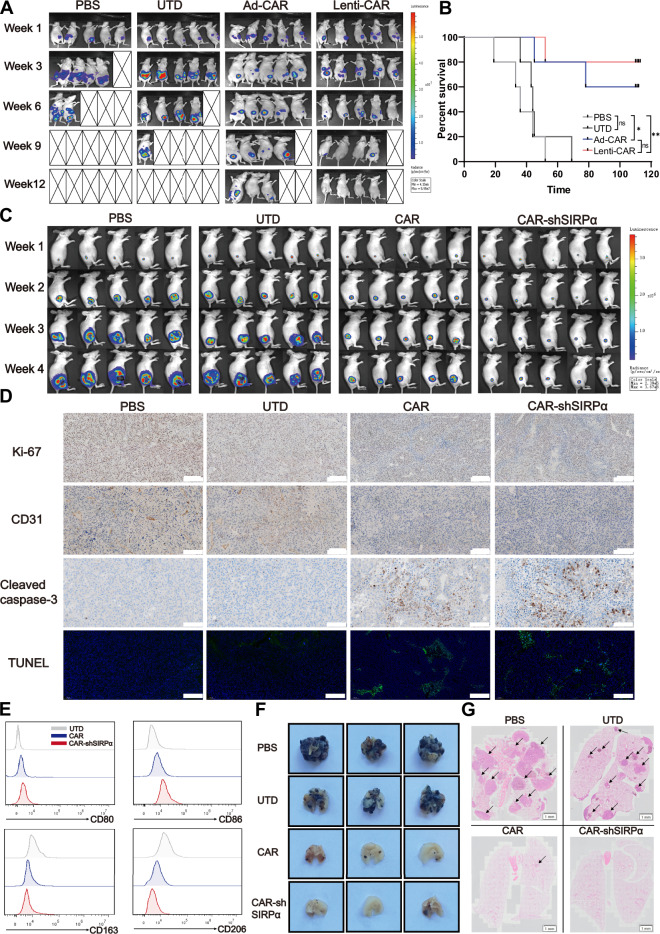
Potent antitumor activity of CAR-shSIRPα macrophages in vivo. **A**, **B** Lenti-CAR and Ad-CAR macrophages were used to treat nude mice bearing peritoneal SKOV3 tumors. **A** The tumor burden was assessed via bioluminescence imaging (BLI), and representative images at different time points were presented. **B** Kaplan‒Meier curve showing the survival of the mice (*n* = 5 mice per group). **C** The tumor burden in the B16-HER2 cell subcutaneous injection model was evaluated via BLI, and representative images at different time points were shown (*n* = 5 mice per group). **D** IHC staining and TUNEL immunofluorescence staining were performed on tumor tissue sections from the B16-HER2 cell subcutaneous injection mouse model to examine the expression of Ki67, CD31, and active caspase-3, as well as apoptosis, in the tumor tissues. Scale bars represent 100 μm. **E** Polarization markers for M1 (CD80 and CD86) and M2 (CD163 and CD206) infiltrating macrophages were analyzed via FACS. **F**, **G** Tumor-bearing mice were euthanized four weeks after receiving caudal vein transfusions of CAR-modified macrophages in the B16-HER2 lung metastasis model. **F** Representative macroscopic images of lungs excised from the specified treatment groups at the end of the experiment. **G** Lung metastatic burden was assessed via HE staining. Scale bars represent 1 mm (*n* = 7 mice per group). For all panels, *P < 0.05, **P < 0.01, ***P < 0.001

To evaluate the efficacy of CAR-modified macrophages in vivo, various tumor models have been established in nude mice. Initially, an intraperitoneal tumor model was developed using SKOV3 cells. One week after implantation, the mice were randomly divided into four treatment groups: the PBS group, the UTD control group, the Ad-CAR group (generated through adenovirus infection), and the Lenti-CAR group (generated through lentivirus infection). Live imaging examinations were conducted on tumor-bearing mice at 1, 3, 6, 9, and 12 weeks posttumor initiation (Supplementary Fig. 8a, b). Tumors exhibited rapid growth in the PBS and UTD treatment groups. In the PBS group, more than half of the mice died within five weeks of tumor growth, whereas 80% of the tumor-bearing mice in the UTD group did not survive beyond 10 weeks. In contrast, substantial growth inhibition of intraperitoneal tumors was observed in the Ad-CAR and Lenti-CAR treatment groups, with only 20% and 10% of the mice, respectively, surviving within ten weeks of tumor development (Fig. 6A). At 100 days posttumor inoculation, 20% and 40% of the mice in the respective treatment groups survived. Statistical analysis revealed a slightly greater therapeutic effect of Lenti-CAR macrophages than of Ad-CAR macrophages (Fig. 6B). Consequently, lentivirus-infected macrophages were used in subsequent in vivo experiments.

Subcutaneous tumor models were created in nude mice via B16-HER2 or ID8-HER2 tumor cells. One week after tumor establishment, the mice were administered PBS, UTD, CAR-M, or CAR-shSIRPα-M via intravenous injection (Supplementary Fig. 8c, d). In vivo imaging over four consecutive weeks in the B16-HER2 tumor model revealed notable inhibition of tumor growth attributed to both CAR-M and CAR-shSIRPα-M. Notably, CAR-shSIRPα-M exhibited the most promising therapeutic effects among the treatment groups (Fig. 6C and Supplementary Fig. 8e). Similar results were observed in the ID8-HER2 tumor model (Supplementary Fig. 8f–h). Immunohistochemical analysis of tumor tissues revealed high levels of Ki67 and CD31 in the PBS and UTD treatment groups, indicating increased tumor proliferation and angiogenesis. Conversely, reduced expression levels of Ki67 and CD31 were observed in the groups treated with CAR and CAR-shSIRPα macrophages (Fig. 6D). Furthermore, few apoptotic cells were detected in the tumor tissues of the PBS and UTD treatment groups. In contrast, significant cleaved caspase-3 staining and TUNEL-positive apoptotic cells were observed in the groups treated with CAR-modified macrophages, particularly in those treated with CAR-shSIRPα macrophages (Fig. 6D). Additionally, mice in the PBS and UTD groups presented significant weight increases due to tumor growth, whereas the weight changes in the CAR and CAR-shSIRPα macrophage-treated groups were not statistically significant (Supplementary Fig. 9a). By the end of the treatment, no apparent abnormalities were noted in the vital organs of the nude mice across all groups (Supplementary Fig. 9b), indicating that the in vivo use of CAR macrophages is safe.

To evaluate the phenotype of the macrophages infiltrating the tumor tissue, we isolated the tumor tissue five days after the intravenous injection of the macrophages and analyzed the polarization markers of the GFP-positive macrophages. Our analysis revealed that, compared with those in the UTD group, infiltrating CAR- and CAR-shSIRPα-treated macrophages presented increased levels of the M1 polarization markers CD80 and CD86, whereas the expression of the M2 polarization markers CD163 and CD206 was significantly reduced (Fig. 6E). This M1 phenotype was particularly pronounced in CAR-shSIRPα-treated macrophages, suggesting that the knockdown of SIRPα may help sustain the M1-polarized state of CAR-Ms in vivo.

To establish a nude mouse model of lung metastasis, B16-HER2 cells were injected intravenously via the tail vein for seven days. Subsequently, the mice were injected with PBS, UTD, CAR macrophages, or CAR-shSIRPα macrophages. After three weeks, the mice were euthanized, and their lung tissues were dissected for observation (Supplementary Fig. 10a, b). Widespread melanized tumor cells were observed on the lung surfaces of the mice in the PBS and UTD treatment groups, whereas only limited lung tumor metastases were present in the CAR-modified macrophage treatment group, with virtually no metastases in the CAR-shSIRPα macrophage treatment group (Fig. 6F). Consistent results were obtained upon examination of lung tissue sections via HE staining (Fig. 6G). Additionally, while there were no significant differences in body weight among the groups during the treatment period (Supplementary Fig. 10c), the ratio of lung weight to body weight was significantly lower in the CAR and CAR-shSIRPα macrophage treatment groups than in the control groups (Supplementary Fig. 10d). Overall, these findings demonstrated a favorable in vivo therapeutic effect of CAR macrophages, which can be further enhanced through the suppression of SIRPα expression.

### CAR-modified macrophages promote functional T-cell tumor infiltration

**Fig. 7:**
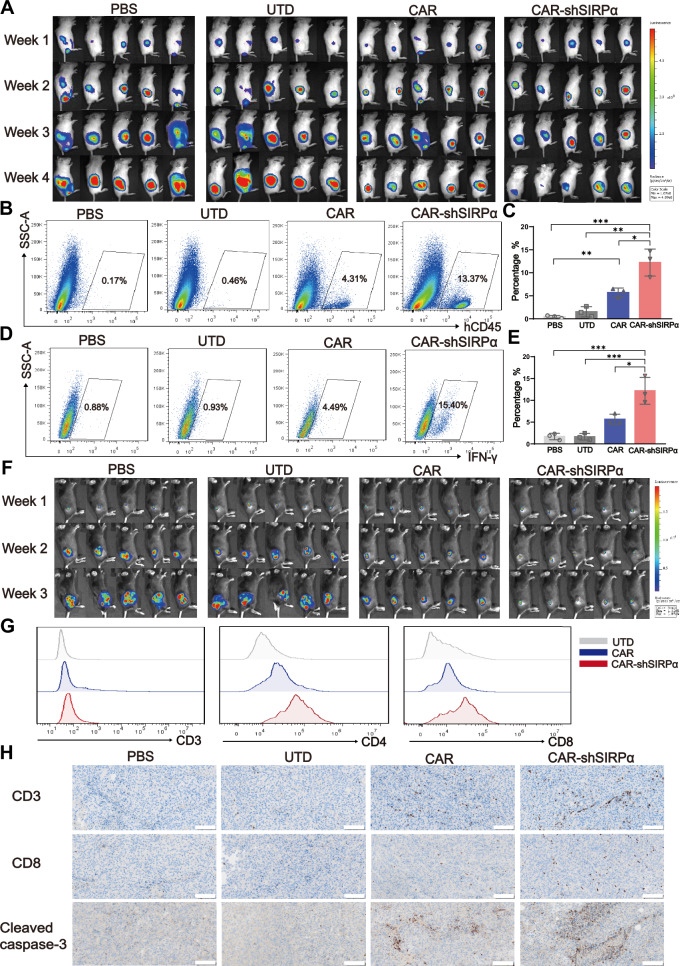
CAR-shSIRPα macrophages promote T-cell infiltration and antitumor immunity. **A** The tumor burden in humanized immune system (HIS) mice treated with CAR-modified macrophages was assessed via BLI, with representative images presented at different time points following tumor implantation (*n* = 5 mice per group). **B**, **C** The percentage of hCD45^+^ cells in the tumor tissues of tumor-bearing HIS mice was measured 2 weeks after treatment, with each point representing one mouse (*n* = 3 per group). **D**, **E** The percentage of IFN-γ^+^ cells among hCD8^+^ T cells in the tumor tissue of tumor-bearing HIS mice was assessed 2 weeks posttreatment, with each point representing one mouse (*n* = 3 per group). **F** The tumor burden in immunocompetent C57BL/6 mice treated with CAR-modified macrophages was evaluated via BLI, with representative images shown at different time points (*n* = 5 mice per group). **G** CD3^+^ T, CD4^+^ T, and CD8^+^ T cells were detected via FACS in tumor tissues from tumor-bearing C57BL/6 mice. **H** IHC staining of CD3, CD8, and active caspase-3 was conducted on tumor tissue sections from tumor-bearing C57BL/6 mice. Scale bars represent 100 μm. For all panels, *P < 0.05, **P < 0.01, ***P < 0.001

Humanized mouse models of cancer, which involve immunodeficient mice coengrafted with human tumors and immune cells, serve as vital tools in immuno-oncology research with potential clinical applications. We established a human immune system (HIS) mouse model by intravenously injecting human peripheral blood mononuclear cells (hu-PBMCs) into NCG mice (Supplementary Fig. 11a). Flow cytometric analysis performed two weeks after hu-PBMC injection revealed an average of 23.98% CD45^+^ cells and 19.05% CD3^+^ cells, indicating that T cells were the predominant component involved in the immune system reconstitution of HIS mice (Supplementary Fig. 11b, c). Following hu-PBMC engraftment for one week, HER2-positive APSC1 cells were subcutaneously transplanted into NCG mice. The adoptive transfer of CAR-modified macrophages was performed two weeks after tumor inoculation, and hCD45^+^ and hCD3^+^ T cells in the peripheral blood of tumor-bearing HIS mice were analyzed two weeks after CAR-modified macrophage treatment (Supplementary Fig. 11d–f). In vivo imaging revealed comparable tumor volumes among all groups before CAR macrophage treatment; however, notable tumor growth inhibition was evident after two weeks of treatment in the mice that received CAR macrophages and those that received CAR-shSIRPα macrophages (Fig. 7A). Four weeks after tumor formation, HIS mice were euthanized, and flow cytometric analysis of tumor tissues revealed minimal infiltration of CD45^+^ cells in the PBS- and UTD-treated groups, in contrast to the substantial increase in CD45^+^ cell infiltration in the groups treated with CAR-modified macrophages, particularly in the CAR-shSIRPα-treated group (Fig. 7B, C). Furthermore, the CAR-shSIRPα-treated group presented the highest proportion of IFN-γ^+^ cytotoxic T cells in the tumor tissues (Fig. 7D, E). Immunofluorescence staining and flow cytometry of tumor tissues revealed significantly greater numbers of CD3^+^ and CD8^+^ cells in both the CAR- and CAR-shSIRPα macrophage-treated groups than in the PBS- or UTD-treated groups, along with decreased Ki67 expression and increased apoptotic cell counts within the tumor tissues (Supplementary Fig. 12).

To analyze the distribution and function of CAR-modified macrophages in vivo more comprehensively, we constructed mouse CAR-Ms and CAR-shSIRPα-Ms via the murine iBMDM cell line. Given that mice do not express FcγRIIa, we utilized FcεRIγ as the intracellular domain to construct a murine CAR. For the experiments assessing the distribution of CAR-modified macrophages in vivo, we created CAR-Luc2 and CAR-shSIRPα-Luc2 constructs, which express luciferase 2 fused with the CAR (Supplementary Fig. 13a). In B16-HER2 subcutaneous tumor-bearing mice, we monitored the distribution of CAR-Luc2 and CAR-shSIRPα-Luc2 macrophages in major organs and tumors after tail vein injection. Our findings revealed that CAR-Luc2 and CAR-shSIRPα-Luc2 macrophages were enriched primarily in the lungs during the first three days, with minimal distribution in the liver. By day five postinjection, these cells had migrated to the liver, and some were present in the tumor tissue. Over time, an increasing number of CAR-Luc2 and CAR-shSIRPα-Luc2 macrophages were found in the tumor tissue. At 15 days after tail vein injection, CAR macrophages were still detectable in the tumor tissue and liver but were consistently undetectable in the spleen or kidneys (Supplementary Fig. 13b).

To further substantiate the promoting effect of CAR-modified macrophages on T-cell tumor infiltration, we established a B16-HER2 subcutaneous tumor model in immunocompetent C57BL/6 mice, and CAR-modified iBMDMs were administered intravenously one week after tumor inoculation. Consistent with the results in the nude mouse models, both CAR- and CAR-shSIRPα iBMDMs significantly inhibited tumor cell growth compared with the PBS and UTD controls, with CAR-shSIRPα iBMDMs demonstrating a more pronounced therapeutic effect (Fig. 7F). Importantly, in the tumor tissues of the mice treated with CAR-shSIRPα iBMDMs, we detected increased numbers of CD3-positive and CD8-positive T cells, along with increased expression of activated caspase-3 (Fig. 7G, H). These findings align with the experimental results from the HIS mouse tumor model, indicating that CAR-shSIRPα macrophages can enhance tumor-killing ability by promoting T-cell infiltration. Overall, these results provide compelling evidence that targeting SIRPα in CAR-modified macrophages not only augments their antitumor efficacy but also fosters a more robust immune response through T-cell recruitment in the tumor microenvironment.

## Discussion

Canonical FcγRs are categorized as either activating or inhibitory on the basis of their intracellular signaling properties. In humans, activated FcγRs include FcγRI, FcγRIIa, FcγRIIc, and FcγRIIIa, each containing ITAMs crucial for receptor expression, surface assembly, and signaling [20]. These ITAMs are situated in the ligand-binding α-chains of FcγRIIa and FcγRIIc or in the signaling γ-chains of FcγRI and FcγRIIIa. In contrast, the inhibitory FcγRIIb exerts its effects through an immunoreceptor tyrosine inhibitory motif (ITIM) in its cytoplasmic region. When FcγR interacts with the IgG Fc fragment, it triggers receptor clustering and aggregation, leading to the phosphorylation of ITAM domains by SRC family kinases, followed by the recruitment and activation of SYK family kinases [35, 36]. The activation of PI3K by SYK is critical for recruiting phospholipase C gamma (PLCγ) and generating inositol trisphosphate (IP3), which mobilizes intracellular Ca2^+^ from the endoplasmic reticulum [37]. These intracellular biochemical changes, along with the subsequent activation of the Rho GTPases CDC2, RAC1, and RAC2 and actin polymerization mediated by the ARP2/3 and WASP proteins, culminate in the phagocytosis of IgG complexes and receptor internalization [38]. Following these early events, various signaling pathways, including the MEK and MAP kinases and the RAS pathway, are activated, leading to the expression of proinflammatory cytokines and chemokines [39–41]. Overall, the phagocytic and proinflammatory functions mediated by FcγRs provide a robust foundation for developing CAR-Ms with potent antitumor capabilities. In this study, we constructed CAR molecules by combining a HER2-targeting scFv with various FcγR-containing ITAMs. Phagocytosis experiments demonstrated that CAR incorporating FcγRIIa displayed superior phagocytic efficiency. FcγRIIa is widely expressed on professional phagocytes, such as monocytes, macrophages, neutrophils, and dendritic cells, and serves as the primary FcγR involved in ADCP [42, 43], a critical and clinically relevant mechanism of action for anticancer monoclonal antibodies [44, 45]. Our findings demonstrated that CAR with FcγRIIa effectively harnesses the tumor-targeting ability of scFv and that the phagocytic impact facilitated by FcγRIIa significantly enhances macrophage phagocytosis and cytotoxicity against tumor cells.

The phagocytic process is finely tuned by the balance of “eat-me” and “don’t-eat-me” signals. Priming macrophages to increase their capacity to engulf cancer cells or blocking negative checkpoints and their ligands that inhibit phagocytosis has emerged as a promising strategy to elicit robust tumoricidal functions in macrophages. In our study, we demonstrated that CAR-containing FcγRIIa transmitted an “eat-me” signal, significantly increasing CAR-M phagocytosis. However, we also observed a marked increase in SIRPα expression in CAR-M cells following coincubation with tumor cells (Fig. 1H). This finding suggested that tumor cells employ additional layers of CD47-SIRPα “don’t-eat-me” signals, mimicking normal cells to deceive CAR-Ms and evade phagocytosis. To counteract the phagocytic inhibition caused by SIRPα upregulation in CAR-M cells, we introduced a specific shRNA to silence SIRPα, which resulted in improved phagocytosis and enhanced the tumor-killing efficiency of CAR-shSIRPα macrophages. Unlike previous reports in which monoclonal antibodies were used to block CD47 or SIRPα, the knockdown of SIRPα in CAR-M cells did not destroy red blood cells, as observed with anti-CD47 antibodies, nor did it cause neurotoxicity in neurons with high SIRPα levels, making it a safer and more reliable strategy.

In antigen-presenting cells, enhanced phagocytosis of tumor cells activates innate immune response pathways. The degradation processes within phagolysosomes allow for the recognition of tumor-derived damage-associated molecular patterns (DAMPs), such as nuclear DNA (nDNA) and single-stranded RNA (ssRNA), by TLRs [22, 46]. This recognition leads to activation of the NF-κB signaling pathway [47]. Our data indicate that CAR-modified macrophages exhibit marked NF-κB activation after engulfing tumor cells, leading to significant upregulation of the proinflammatory cytokines IL-1 and TNF-α. Additionally, SIRPα inhibition potentiated the inflammatory response elicited by CAR-Ms, as shown in Figs. 2K, M, 4H–K, and 5A–C. Intriguingly, we also detected activation of the cGAS-STING signaling pathway in CAR-Ms and SIRPα-silenced CAR-Ms upon coculture with tumor cells (Fig. 5G). The cGAS-STING pathway, a key component of the innate immune system, detects cytosolic DNA from pathogens or self-DNA released during cellular stress or damage, stimulating the transcription of proinflammatory cytokine genes, including type I interferons and TNF-α, through the phosphorylation of IRF3 and NF-κB [22]. Previous studies have demonstrated that blocking CD47 significantly enhances CD103^+^ dendritic cell absorption of tumor DNA, activating the cGAS-STING pathway [48]. Another study revealed that mitochondrial DNA (mtDNA), rather than nuclear DNA (nDNA), escapes from phagolysosomes into the cytosol and activates the cGAS pathway [49]. Importantly, our current study is the first to confirm cGAS-STING pathway activation in CAR-modified macrophages, demonstrating that SIRPα inhibition significantly enhances this activation, contributing to our understanding of the antitumor effects of CAR-modified macrophages.

In addition to tumor-specific phagocytic effects, we identified a nonspecific cytotoxic response induced by CAR-Ms in vitro (Fig. 3G, I). Coculture with CAR-modified macrophages increased apoptosis in nonphagocytosed tumor cells, and SIRPα inhibition further augmented this effect. Transcriptome sequencing revealed notable upregulation of ROS signaling following SIRPα inhibition, indicating an intensified respiratory burst and substantial ROS production by CAR-shSIRPα-Ms. Previous research has indicated that SIRPα not only limits phagocytosis but also serves as an inhibitory regulator of the NADPH oxidase NOX2 (gp91), mitigating cellular damage from ROS [50]. Consistent with these findings, we observed a significant increase in the expression of genes related to the NADPH oxidase complex, including NOX2, NOX1, NOX3, NCF1, NCF2, RAC1, and RAC2, in CAR-modified macrophages following coculture with tumor cells. This effect was more pronounced in CAR-shSIRPα-treated macrophages (Fig. 5J). Coculture also revealed that decreased SIRPα expression was correlated with increased iNOS expression and NO production (Fig. 5K, L). Consequently, the generation of ROS and NO, which are powerful oxidizing agents, amplifies the anticancer capabilities of CAR-modified macrophages.

Macrophages function not only as professional phagocytes but also as antigen-presenting cells. When tumor cells degrade in lysosomes, tumor-derived antigen peptides are presented on major histocompatibility complexes (MHCs), facilitating T-cell cross-presentation, activation, and priming, thereby linking the innate and adaptive immune responses. While the initial antitumor effects of CAR-modified macrophages primarily stem from direct tumor phagocytosis, the adaptive immune response—especially that of CD8^+^ T cells—plays a crucial role in sustained tumor suppression. In experiments involving both HIS mice and immunocompetent C57BL/6 mice, we observed a significant increase in T-cell infiltration in cohorts treated with CAR-modified macrophages compared with the control group. Inhibition of SIRPα augmented the presence of IFN-γ^+^ cytotoxic T cells within the tumor tissue, resulting in enhanced tumor cell eradication (Fig. 7). However, the potential of CAR-modified macrophages with SIRPα blockade to improve antigen presentation, elicit a more durable memory T-cell response, and affect the tumor microenvironment warrants further investigation.

## Materials and methods

### Cell lines

Human THP-1, SKOV3, SKBR3, DLD-1, and ASPC1 tumor cell lines and mouse-derived B16, ID8, and MC38 cell lines were acquired from Procell Life Science and Technology Co., Ltd., Wuhan, China. The cell lines were cultured in RPMI-1640 or DMEM supplemented with 10% FBS, 1% penicillin‒streptomycin, 1× glutamine, and 1x HEPES at 37 °C in a 5% CO_2_ incubator. All the tumor cell lines were transduced with a lentiviral vector that coencoded luciferase under the CMV promoter and mCherry-T2A-Puro under the EF1α promoter. The transduced target cell lines were selected via puromycin before being used as targets in both the in vitro and in vivo experiments. Similarly, DLD-1, B16, ID8, and MC38 cell lines were infected with the lentivirus pCDH-hHER2t-T2A-BSD. This lentivirus encodes blasticidin and a truncated version of human HER2 without an intracellular domain. Blasticidin selection was performed to obtain HER2-positive transduced cell lines. THP-1-derived macrophages were obtained by differentiating THP-1 cells with 20 ng/ml PMA for three days, followed by a two-day rest in complete RPMI-1640 culture medium.

### Plasmid construction and virus production

For lentivirus production, CAR constructs were cloned and inserted into a third-generation lentiviral backbone via standard molecular biological techniques. The CAR constructs consisted of a CD8 leader sequence, the scFv of P1h3 or FMC63, and the transmembrane and intracellular domains of FcεRIγ, FcγRIIa, or FcγRIIc, which were expressed under the control of the SFFV promoter. All the constructs included a GFP gene driven by the PGK promoter. To detect the distribution of CAR-modified macrophages in vivo, CAR-Luc2 and CAR-shSIRPα-Luc2 were constructed by replacing GFP with luciferase 2. To suppress SIRPα expression, the lentiviral vector CAR-shSIRPα was constructed via a miR-30-based shRNA cassette downstream of the GFP or luciferase 2 gene. Lentiviruses were generated in HEK293T cells, and subsequent purification and concentration steps were performed according to previously described protocols. The production, expansion, concentration, and purification of the replication-deficient Ad5f35 adenovirus were conducted by Beijing Fiveplus Gene Technology Co., Ltd.

### Statistical analysis

GraphPad Prism 9.0 software was used to conduct the statistical analyses. All the data were expressed as the means ± standard error of the means (means ± s.e.m.). ANOVA multiple-comparison *P* values were generated via Tukey’s multiple-comparisons test. All *t* tests were two-sided unless otherwise indicated. For all figures, * indicates *P* < 0.05, ** indicates *P* < 0.01, and *** indicates *P* < 0.001.

## Supplementary information

SUPPLEMENTAL MATERIAL

Original image of WB
