# Supplementary material for: Silencing of SIRPα enhances the antitumor efficacy of CAR-M in solid tumors
Source: Cell Mol Immunol. 2024 Oct 8;21(11):1335–49. doi: 10.1038/s41423-024-01220-3 (PMC11527885; doi:10.1038/s41423-024-01220-3)
Supplement: Supplementary file 1 — SUPPLEMENTAL MATERIAL [file 41423_2024_1220_MOESM1_ESM.docx]

Supplementary Materials for

Silencing of SIRPα enhances the antitumor efficacy of CAR-M in solid tumors

Han Zhang, Yi Huo, Wenjing Zheng, Peng Li, Hui Li, Lingling Zhang, Longqi Sa, Yang He,

Zihao Zhao, Changhong Shi, Lequn Shan, Angang Yang, Tao Wang

**This file includes:**

Supplementary Materials and Methods

Supplementary Figures 1 to 13

**Supplementary Materials and Methods**

**Flow cytometry**

THP-1-derived macrophages were tested for CAR expression using a two-step staining protocol: human HER2/ERBB2 protein-His tag (10004-H08H-100, Sino Biological), TruStain FcX (422302, BioLegend), and anti-His Tag APC (362605, BioLegend). TruStain FcX was used for fluorescence-activated cell sorting (FACS) staining of macrophages expressing Fc receptors. Macrophage purity was tested using Anti-CD11b APC-Cy7 (301341, Biolegend) Anti-CD14 APC (301808, BioLegend). M1 and M2 markers on human macrophages were detected using the following panel: Anti-CD80 PE (12-0809-42, Invitrogen), Anti-CD86 APC (17-0869-42, Invitrogen), anti-HLA-DR PE-Cy7 (25-9956-42, Invitrogen), Anti-CD163 APC (17-1639-42, Invitrogen), Anti-CD206 PE-Cy7 (25-2069-41, Invitrogen). HER2 expression in tumors and SIRPα expression in macrophages were detected using Anti-CD340/HER2 APC (324407, BioLegend) or Anti-CD172/SIRPα APC (323810, BioLegend). Apoptosis was detected using Anti-Annexin V PE (559763; BD Bioscience Pharmingen). The detection of immune cells in humanized mouse peripheral blood is completed by Anti-CD45 FITC (304006, Biolegend), Anti-CD3 APC (300458, Biolegend), Anti-CD8 PE-CY7 (344750, Biolegend) and Anti-IFN-γ FITC (502505, Biolegend) were used to detect the content of immune cells within the tumor. Appropriate fluorescence-matched isotype controls were acquired from the BioLegend. Flow cytometry data were acquired using a BD FACS Canto ™ II (BD Biosciences) or CytoFLEX (Beckman Coulter) and analyzed using FlowJo or Cytexpert.

**FACS-based phagocytosis assay**

FACS-based phagocytosis assay was also performed. 1×105 GFP control or GFP+ CAR-modified macrophages were cocultured with 1×105 mCherry+ B16 cells (HER2−) or 1×105 mCherry+ SKOV3, SKBR3, B16-HER2, MC38-HER2, ID8-HER2, or DLD1-HER2 (HER2+) target cells for 1 h at 37 °C in triplicate. After coculture, the cells were harvested using Accutase (Innovative Cell Technologies) and analyzed using FACS. The percentage of mCherry+ events within the GFP+ population was plotted to represent the phagocytosis rate.

**In vitro cytotoxicity assay**

Luciferase-based killing assays used HER2 and luciferase double-positive SKOV3, SKBR3, and DLD-1-HER2 tumor cells as targets. A standard luciferase-based cytotoxicity test was used to determine the short-term quantitative cytotoxicity of the CAR-modified macrophages. Macrophages were pre-distributed into 96-well plates, and tumor cells were added at various effector-to-target (E:T) ratios after 6 h. Subsequently, the cells were cocultured at 37 °C for 24 h. Luciferase fluorescence values were detected using the IVIS Spectrum (PerkinElmer) to calculate the killing efficiency using the following formula: lysis (%) = [(sample signal–tumor only signal)] / [(background signal–tumor only signal)] × 100.

**Real-time imaging video**

5x105 GFP control macrophages or CAR-modified macrophages were cocultured with 1x105 SKOV3 cells in 12-well plates. Lumascope 720 (Etaluma, Inc.) was used in a 37 °C incubator with 5% CO2. The cell response was imaged continuously for 24 h, at a frame rate of 5 min. Time-lapse images were obtained using a circular fluorescence microscope at 20X magnification.

**Cytokine analysis**

Cytokine levels were determined using ELISA kits. Human IL-1β (Novus Biologicals), TNF-α (Novus Biologicals), IFN-γ (MULTISCIENCES), and mouse IFN-β (Novus Biologicals) ELISA kits were used to measure cytokine levels in cell culture supernatants. All ELISAs were performed in accordance with the manufacturer's instructions.

**Real-time PCR**

RNA extraction was performed using a MiniBEST Universal RNA Extraction Kit (9767, Takara). A total of 1 μg of RNA was reverse-transcribed using PrimeScript II Reverse Transcriptase (2690A, Takara). For real-time PCR analysis, sample cDNA, primers, and TB Green® Fast qPCR Mix (RR430A, Takara) were used according to the manufacturer’s instructions. The following primers were used: CD80, F', AAACTCGCATCTACTGGCAAA, R', GGTTCTTGTACTCGGGCCATA; CD86, F', CTGCTCATCTATACACGGTTACC, R', GGAAACGTCGTACAGTTCTGTG; and TNF, F', GAGGCCAAGCCCTGGTATG, R', CGGGCCGATTGATCTCAGC.

**Western blotting**

Control and CAR-modified macrophages were co-incubated with SKOV3 cells at an E:T ratio of 10:1 for 24 h. Tumor-stimulated macrophage samples were acquired using flow cytometry or EasySep™ immunomagnetic beads (17858, Stem Cell). All proteins were extracted using RIPA lysis buffer (R0010, Solarbio) with a cOmplete™ Protease Inhibitor Cocktail (04693116001, Roche). The proteins were detected using anti-SIRPα (ab191419, Abcam), anti-PFKFB3 (13763-1-AP, Proteintech), anti-LDHA (19987-1-AP, Proteintech), anti-cGAS (26416-1-AP, Proteintech), anti-STING (19851-1-AP, Proteintech), anti-pSTING (Ser366) (50907T, Cell Signaling Technology), anti-IRF3 (66670-1-Ig, Proteintech), anti-pIRF3 (Ser396) (29528-1-AP, Proteintech), anti-iNOS (18985-1-AP, Proteintech), anti-β-actin (4970S, Cell Signaling Technology), and anti-GAPDH (2118S, Cell Signaling Technology) antibodies.

**RNA sequencing**

UTD- and CAR-modified macrophages were cocultured with SKOV3 tumor cells in 10 cm dishes for 24 h at an E:T ratio of 10:1. Macrophages were sorted using flow cytometry. Total RNA extraction, library construction, and RNA sequencing were conducted by NovelBio Bio-Pharm Technology Co., Ltd..

**Organoid culture**

Surgically resected tissues were collected from patients diagnosed with pancreatic or gallbladder cancers. Informed consent was obtained from all the patients enrolled in this study. The tumor tissues were minced and digested using a solution containing 1.5 mg/ml Collagenase II (Sigma-Aldrich), 10 μg/ml Hyaluronidase IV (Sigma-Aldrich), and 10 μM Y-27632 (Sigma-Aldrich). The suspension was then filtered, centrifuged, and the cells were suspended in cold 70% Matrigel and seeded into 24-well culture plates. For passaging, matrigel drops were scraped and digested using TrypLE Express (Gibco). After centrifugation, cell pellets were resuspended in Matrigel and seeded according to the aforementioned procedure. The organoids were propagated roughly every 1-2 weeks, and organoids from the 8th to 15th passages were selected for subsequent experiments.

**Coculture cancer organoids with CAR-modified macrophages**

GFP, CAR, and CAR-shSIRPα macrophages were digested separately using Accutase (Innovative Cell Technologies) to obtain single cells, which were then counted. Prior to coculture, organoid-like structures of varying volumes in matrigel were separated by density gradient centrifugation. Appropriately sized organoids were digested into single cells using TrypLE Express and counted to estimate the number of cells in the tumor organoid clusters. After staining the organoids with 1 μM Cell Trace Red (Invitrogen), GFP, CAR, and CAR-shSIRPα macrophages were individually embedded in 50% Matrigel at an E:T ratio of 10:1. Cocultures were photographed at specified time points to assess the macrophage-mediated destruction of HER2 positive organoids. Some organoids and CAR-M cells were fixed at room temperature using 4% paraformaldehyde and embedded in paraffin blocks for subsequent IHC staining. The supernatant collected from macrophage-organoid cocultures after 72 h was analyzed for the expression levels of IL-1β and TNF-α using ELISA.

**Immunohistochemical and immunofluorescence**

The tissue specimens were fixed in formalin and embedded in paraffin. Subsequently, the tissue sections were dewaxed, hydrated, and subjected to antigen repair, blocking, and staining. For IHC staining, Anti-CD31 (GB113751; Servicebio), Anti-CD3 (GB111337; Servicebio), Anti-CD8 (GB12068; Servicebio), and Anti-Ki67 (GB111499; Servicebio) antibodies were used. Immunofluorescence staining utilized Anti-CD3 (GB12014, Servicebio), Anti-CD8 (GB12068, Servicebio), and TUNNEL (G1501, Servicebio). Data analysis was performed using Caseviewer software.

**Xenograft tumor model in nude mice**

Female BALB/c nude mice or C57BL/6 mice aged 6-8 weeks were obtained from Gempharmatech Co., Ltd. and housed in a pathogen-free (SPF) facility at the Air Force Military Medical University (AFMU). All animal experiments were conducted in accordance with ethical guidelines and were approved by the Animal Use Committee of the AFMU. For the intraperitoneal tumor model, 1x106 SKOV3 tumor cells were injected intraperitoneally into nude mice. One week or six weeks after SKOV3 implantation, 6x106 control or CAR-modified macrophages were injected intraperitoneally into the tumor-bearing mice. Bioluminescence imaging (BLI) using the IVIS imaging system was performed at specified time points to monitor tumor growth, and survival time was recorded. For the subcutaneous tumor model, either 2x105 B16-HER2 cells or 1x106 ID8-HER2 cells were subcutaneously injected into the right flank of the mice. One week after tumor implantation, the mice were randomly divided into four groups. B16-HER2 tumor-bearing mice were injected with PBS, 2x106 UTD, CAR, or CAR-shSIRPα macrophages via tail vein. Simultaneously, ID8-HER2 tumor-bearing mice were injected with PBS, 7x106 UTD, CAR, or CAR-shSIRPα macrophages via the tail vein. Weekly BLI was performed, and tumor volume was calculated using the formula V = (W^2 x L) / 2, where W < L. Tumor-bearing mice were euthanized five weeks after tumor implantation, and tumor sections were subjected to hematoxylin and eosin (HE) staining, as well as immunohistochemistry (IHC) staining. For the lung metastasis model, 2x105 B16-HER2 tumor cells were injected into the tail vein. On day 8, PBS or 4x106 UTD, CAR, or CAR-shSIRPα macrophages were injected into the tail vein of mice. Tumor distribution in the lungs of mice was observed after four weeks, and the lung sections were subjected to HE staining.

**Tumor‑bearing model in NCG mice**

NCG mice, aged 6-8 weeks, were purchased from GemPharmatech Co., Ltd. (Nanjing, China). Human whole peripheral blood mononuclear cells (PBMCs) were isolated using a Lymphoprep (Milestone Biotechnology). The NCG mice were intravenously injected with 1× 107 human PBMCs. On day 7, HER2+ ASPC1 cells (1× 106) were subcutaneously injected into the right flank of NCG mice. On day 14, peripheral blood was collected from the mice via the tail vein for flow cytometric analysis of hCD45+ and hCD3+ cell proportions in PBMCs. Two weeks after tumor implantation, mice were intravenously injected with PBS or 6x106 UTD, CAR, or CAR-shSIRPα macrophages via the tail vein. Weekly BLI was performed for 4 weeks, starting with tumor implantation. Four weeks after tumor implantation, the mice were euthanized and the tumor tissues were isolated and digested into single cells using collagenase II(Sigma). Flow cytometry was used to analyze CD3+ human T cells and IFN-γ+ CD8+ T cells. In addition, some tumor tissues were sliced and the tumor sections were subjected to IHC staining.

**Supplementary Figures 1 to 13**


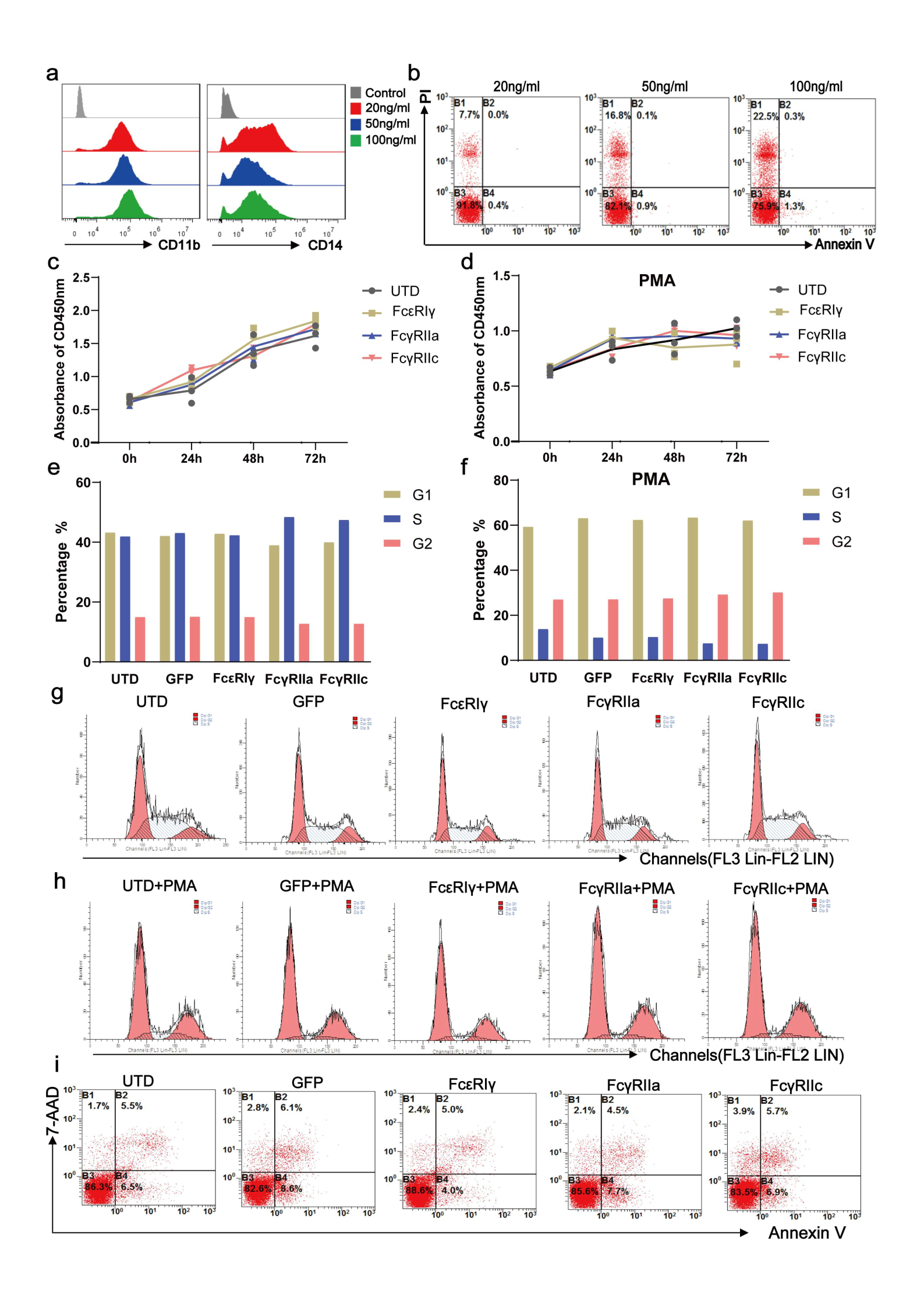


**Supplementary Figure 1: Optimization of THP-1-induced differentiation and the proliferation, cell cycle, and apoptosis of macrophages post-CAR transfection**.

**a**, Flow cytometry was employed to assess the expression levels of CD11b and CD14 in THP-1 differentiated macrophages following stimulation with PMA at concentrations of 20 ng/mL, 50 ng/mL, and 100 ng/mL. **b**, Cell viability of THP-1 differentiated macrophages post-stimulation with PMA at the aforementioned concentrations was determined using flow cytometry. **c**, **d**, The proliferation capability of UTD, P1h3-FcεRIγ, P1h3-FcγRIIa, and P1h3-FcγRIIc cells was assessed before (c) and after (d) differentiation induced by PMA using a CCK-8 assay kit. **e-h**, Flow cytometry was utilized to analyze the cell cycle of UTD, GFP, P1h3-FcεRIγ, P1h3-FcγRIIa, and P1h3-FcγRIIc cells before (e & g) and after (f & h) differentiation induced by PMA. i. The cell viability of UTD, GFP, P1h3-FcεRIγ, P1h3-FcγRIIa, and P1h3-FcγRIIc cells was determined through flow cytometry analysis.


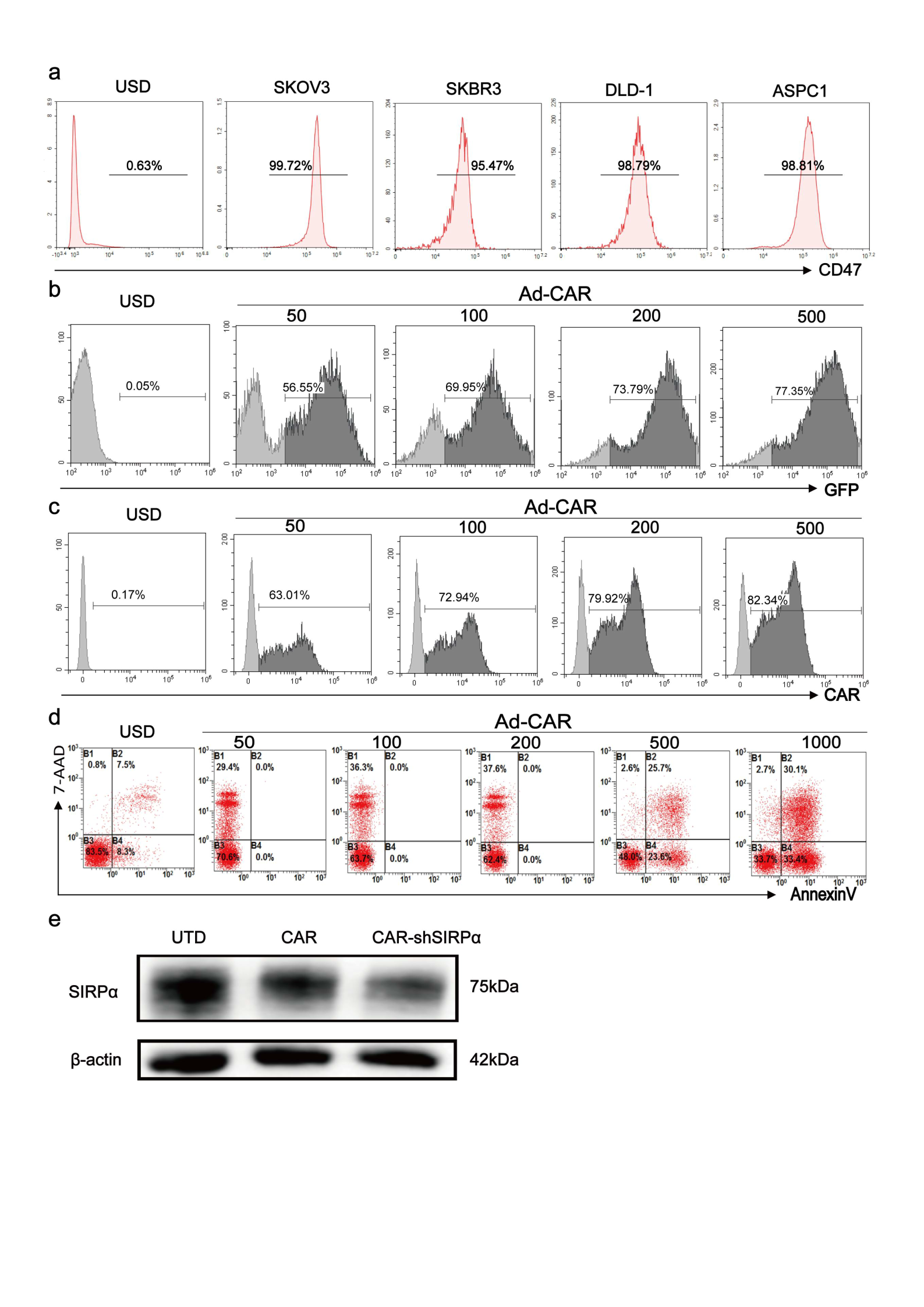


**Supplementary Figure 2: Infection of macrophages with Ad5F35 adenovirus**

**a**, CD47 expression was assessed in SKOV3, SKBR3, DLD-1, and ASPC1 cells using flow cytometry. **b**, **c**, Macrophages were infected with Ad5F35 adenovirus at multiplicities of infection (MOIs) of 50, 100, 200, and 500, and the expression of GFP (b) and CAR molecules (c) was analyzed by flow cytometry to evaluate infection efficiency. **d**, Flow cytometry was employed to evaluate the cellular activity of macrophages infected with Ad5F35 adenovirus at varying MOIs. **e**, SIRPα expression in untreated (UTD) macrophages and macrophages infected with CAR and CAR-shSIRPα via adenovirus was detected using Western blotting.


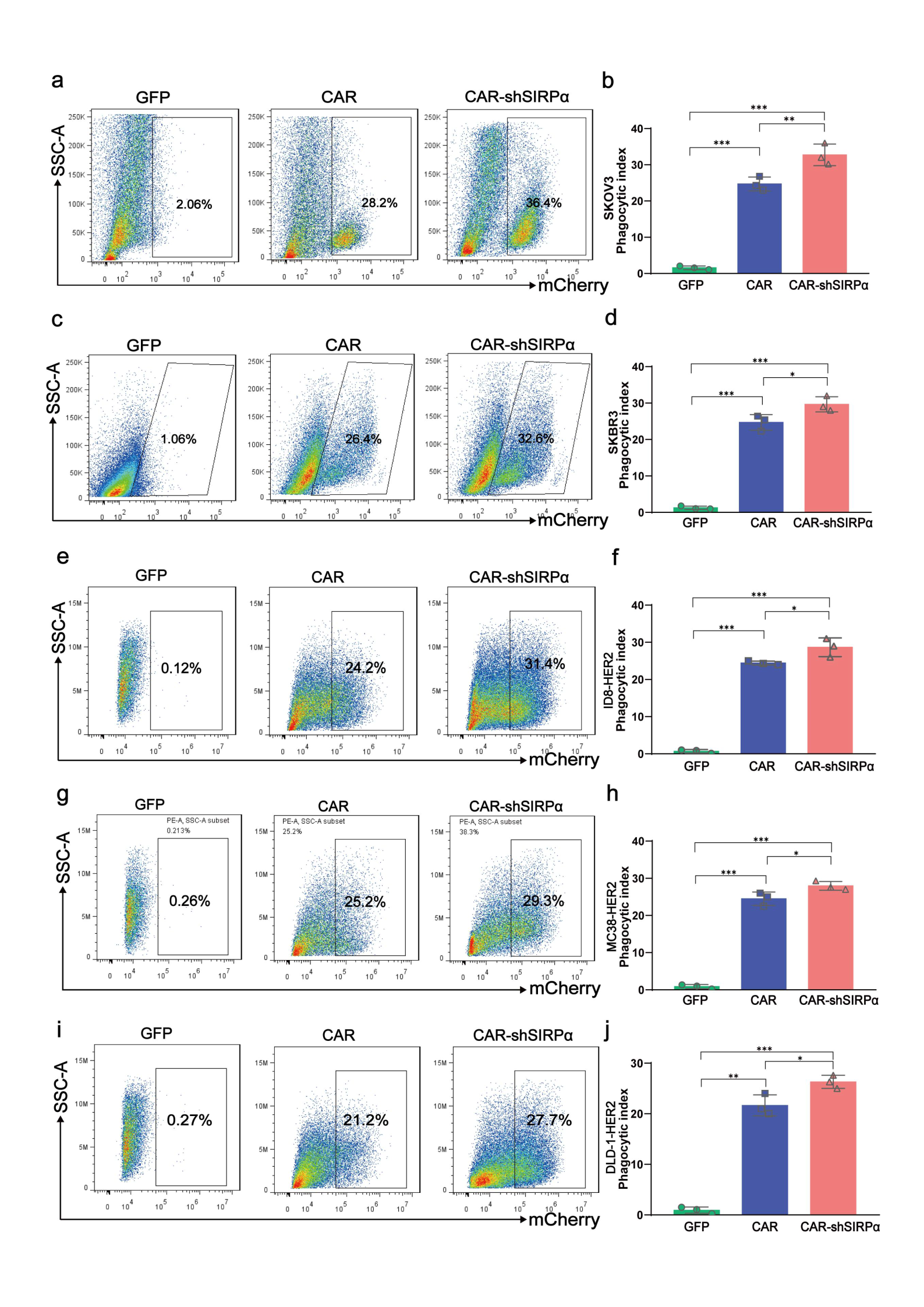


**Supplementary Figure 3: Phagocytosis of GFP-M, CAR-M, and CAR-shSIRPα-M on HER2-positive tumor cells**

Macrophages and tumor cells were co-cultured at a ratio of 1:1 for 1 hour. Phagocytosis of GFP-M, CAR-M, and CAR-shSIRPα-M on SKOV3 (a & b), SKBR3 (c & d), ID8-HER2 (e & f), MC-38-HER2 (g & h), and DLD-1-HER2 (I & j) was detected using flow cytometry.


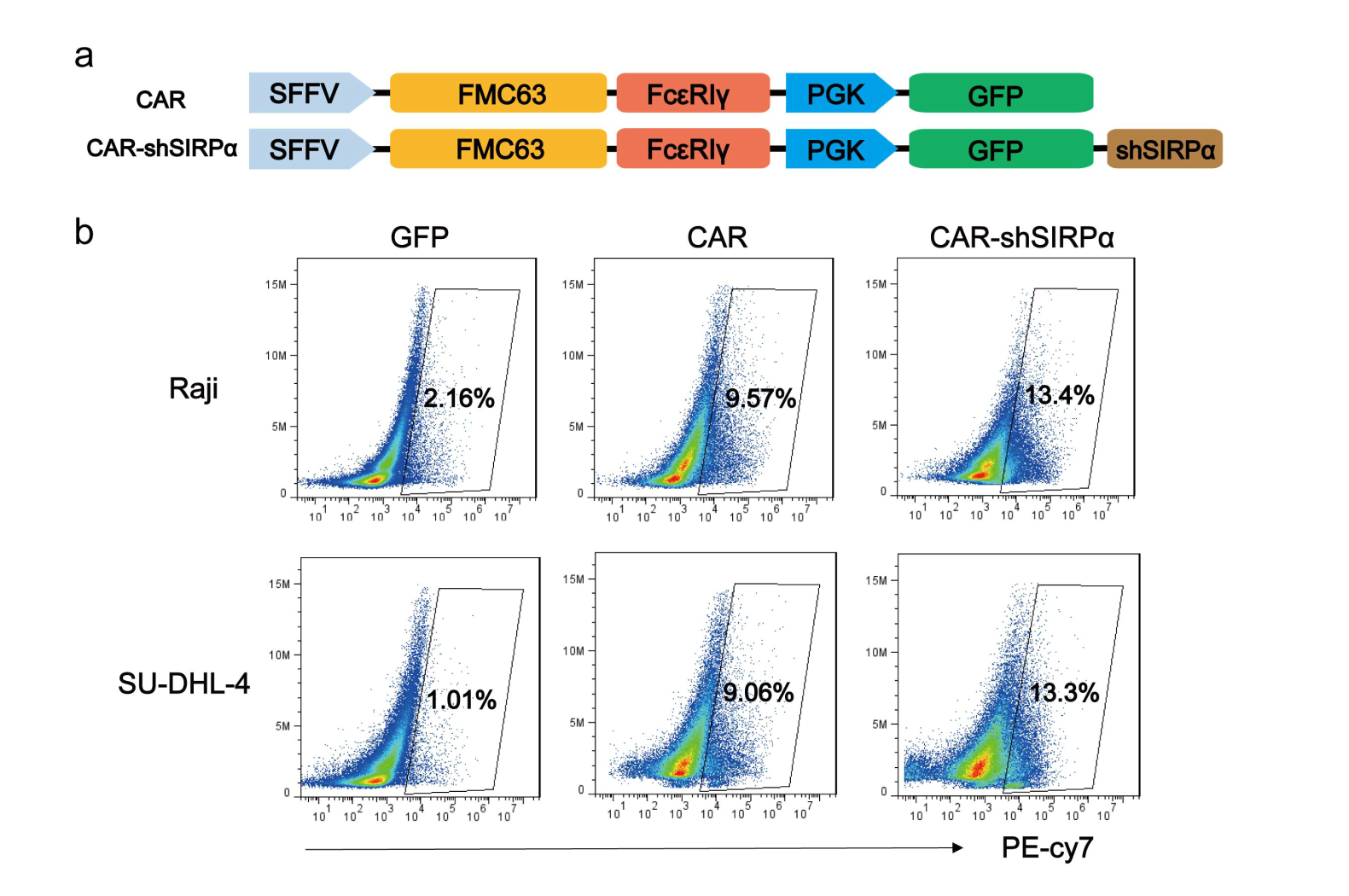


**Supplementary Figure 4: Phagocytosis of CD19-positive tumor cells by CAR-modified macrophages.**

**a**, Structural diagrams of CAR and CAR-shSIRPα macrophages targeting CD19. **b**, Phagocytosis measurements of CD19- and mCherry-positive Raji and SU-DHL-4 cells by CAR and CAR-shSIRPα macrophages following coculture for 1 hour


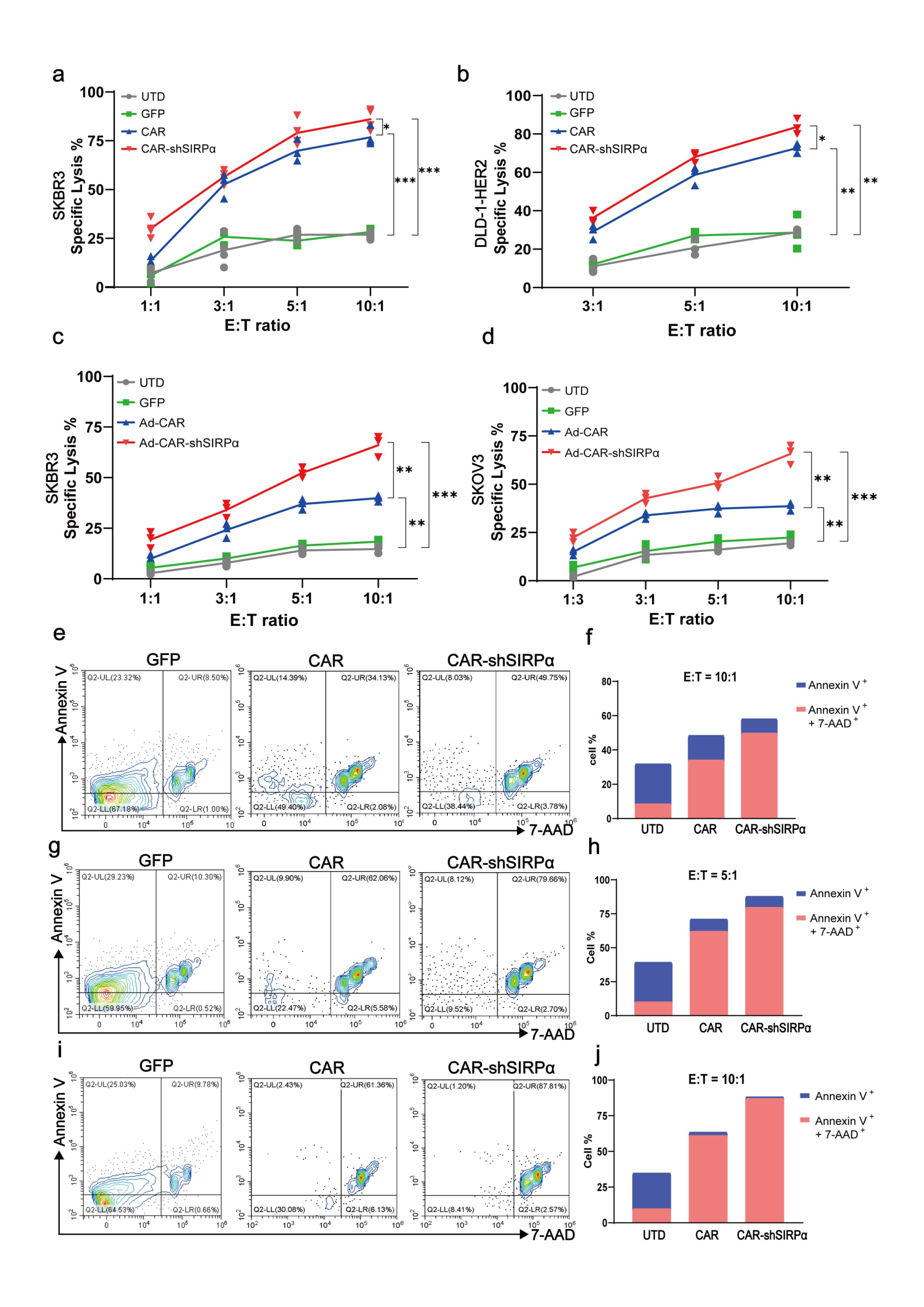


**Supplementary Figure 5: Cytotoxic effects of GFP-M, CAR-M, and CAR-shSIRPα-M on HER2-positive tumor cells**

**a**, **b**, UTD, GFP-M, CAR-M, and CAR-shSIRPα-M were cocultured with SKBR3 (a) and DLD-1-HER2 (b) cells at various effector-to-target ratios for 24 hours. The cytotoxicity of different macrophages against tumor cells was assessed through luciferase expression. **c**, **d**, UTD, Ad-GFP-M, Ad-CAR-M, and Ad-CAR-shSIRPα-M were cocultured with SKBR3 (c) and SKOV3 (d) cells, respectively, at different effector-to-target ratios for 24 hours. The tumor cell killing efficiency of various macrophages was determined by luciferase expression. **e**, **j**, GFP-M, CAR-M, and CAR-shSIRPα-M were cocultured individually with SKOV3 cells at an Effector-to-Target (E:T) ratio of 10:1 for 24 hours (e & f), at an E:T ratio of 5:1 for 48 hours (g & h), or at an E:T ratio of 10:1 for 48 hours (i & j). Subsequently, the activity of non-phagocytosed tumor cells was analyzed using flow cytometry.


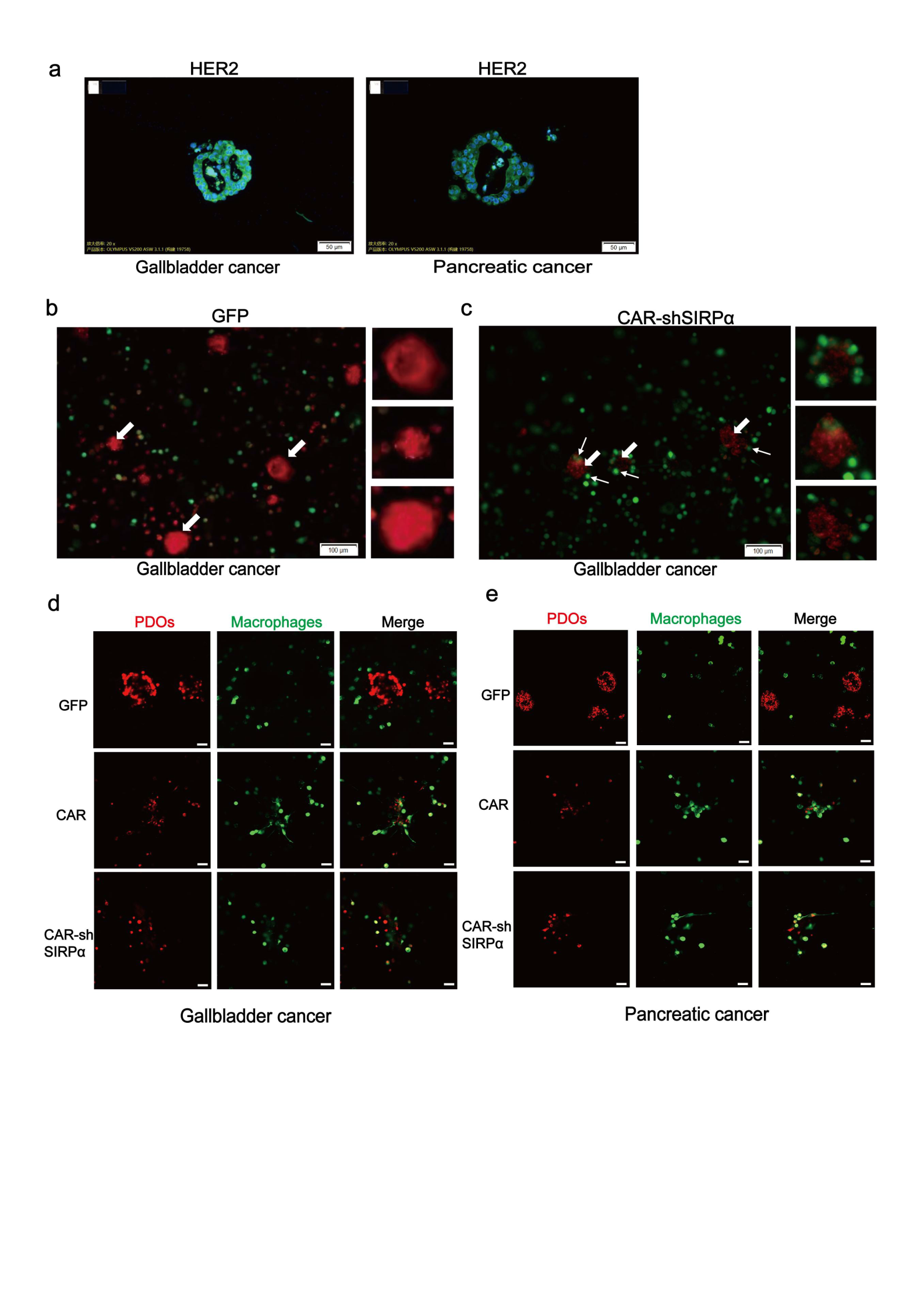


**Supplementary Figure 6: Phagocytosis of patient-derived organoids (PDOs) by CAR-modified macrophages**

**a**, Immunofluorescence analysis of HER2 expression in patient-derived organoids (PDOs) from gallbladder and pancreatic cancers. **b**, **c**, Gallbladder cancer PDOs labeled with Track It™ Red were cocultured with GFP-M (b) or CAR-shSIRPα-M (c) for 24 hours. Fluorescence microscopy was utilized to observe the attachment of macrophages to the periphery of gallbladder cancer PDOs (bold arrows indicate PDOs; thin arrows indicate macrophages). **d**,**e**, Gallbladder cancer PDOs (d) or pancreatic cancer-like PDOs (e), labeled with Track It™ Red, were cocultured with GFP-M, CAR-M, or CAR-shSIRPα-M for 24 hours. Phagocytosis was assessed using confocal microscopy.


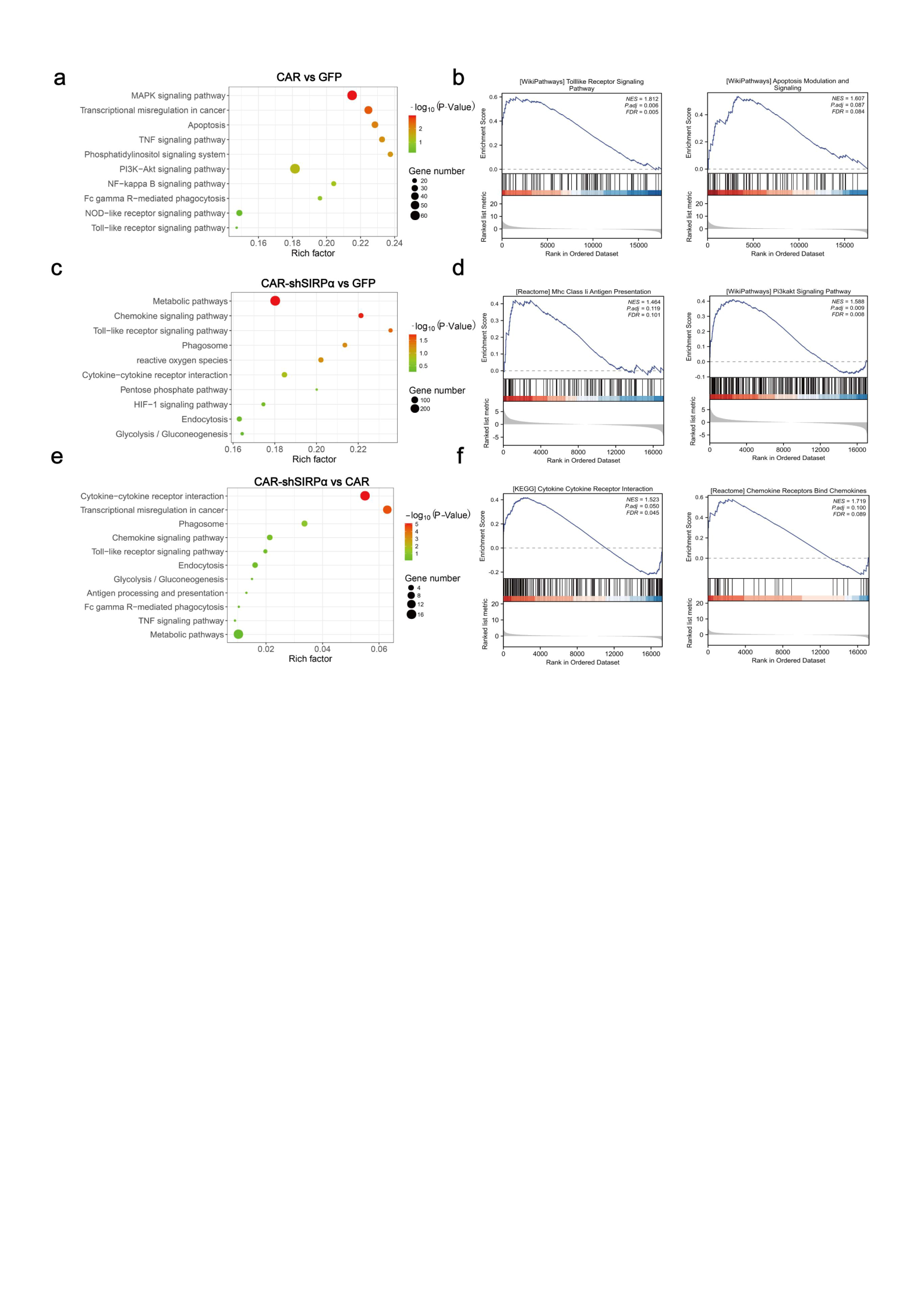


**Supplementary Figure 7: Functional enrichment analysis using transcriptional sequencing data**

SKOV3 cells cocultured with GFP, CAR, or CAR-shSIRPα macrophages were sorted using flow cytometry and subsequently subjected to RNA sequencing. Gene Set Enrichment Analysis (GSEA) was conducted on the top 100 upregulated differentially expressed genes (DEGs) comparing CAR-M vs. GFP-M (a), CAR-shSIRPα-M vs. GFP-M (c), and CAR-shSIRPα-M vs. CAR-M (e). Additionally, Kyoto Encyclopedia of Genes and Genomes (KEGG) pathway enrichment analysis of the DEGs was performed, highlighting representative pathways for CAR vs. GFP (b), CAR-shSIRPα vs. GFP (d), and CAR-shSIRPα vs. CAR (f).


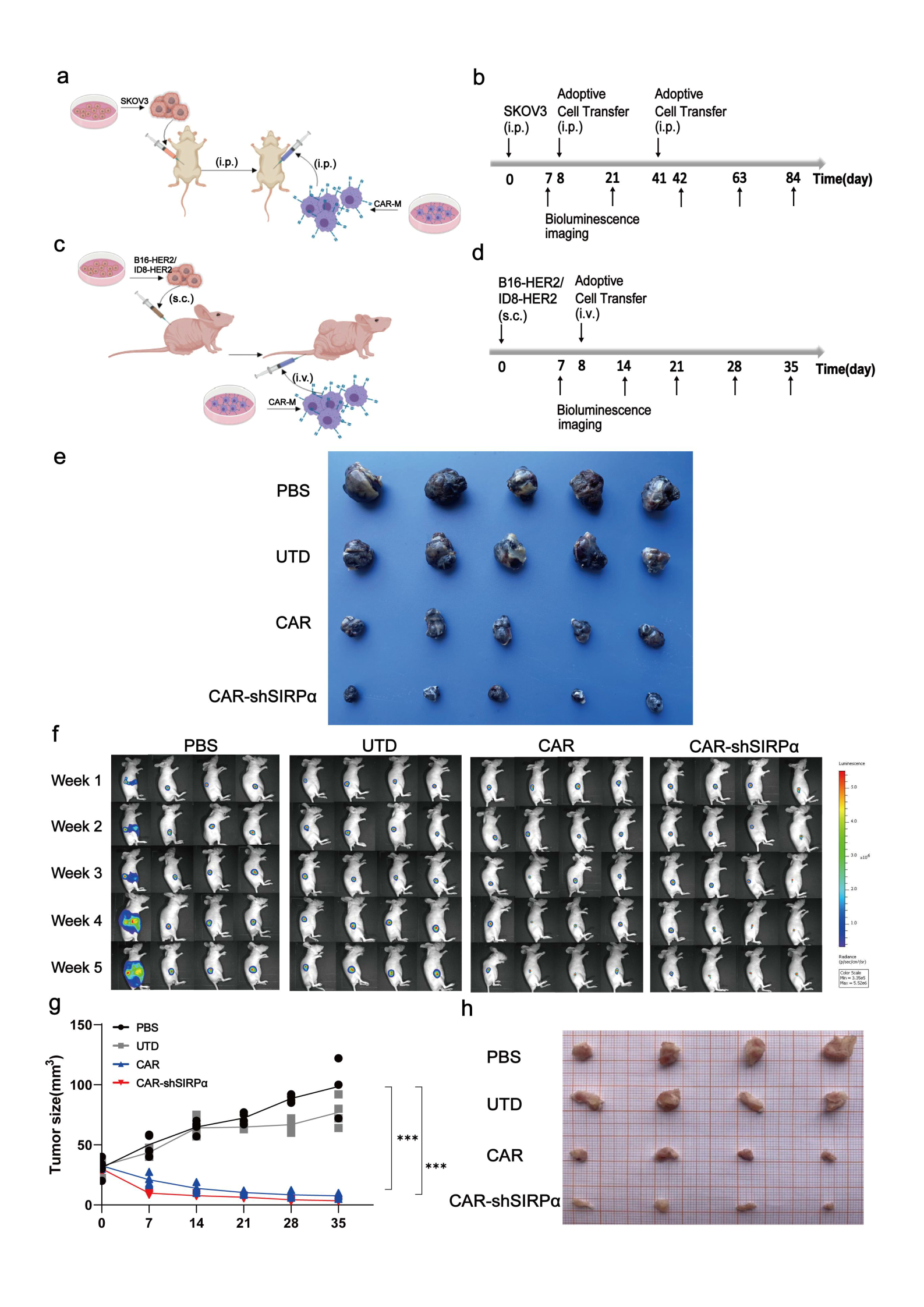


**Supplementary Figure 8: Inhibition of SIRPα enhances CAR-Mediated antitumor effects *in vivo***

**a**,**b**, Establishment and treatment of a peritoneal tumor cell-bearing model in nude mice. (a) Schematic diagram. (b) Timeline of relevant procedures. **c**, **d**, Establishment and treatment of a subcutaneous tumor cell-bearing model in nude mice. (c) Schematic diagram. (d) Timeline of relevant procedures. **e**, Tumors isolated from B16-HER tumor-bearing nude mice after three weeks of CAR-modified macrophage treatment. **f**, The tumor burden in subcutaneous ID8-HER2 tumor-bearing nude mice treated with CAR-modified macrophages was evaluated using bioluminescence imaging (BLI), with representative images captured at different time points (n = 4 mice per group). **g**, Measurements of tumor volume in subcutaneous ID8-HER2 tumor-bearing mice during CAR-modified macrophage treatment. **h**, Tumors isolated from ID8-HER2 tumor-bearing mice after five weeks of CAR-modified macrophage treatment.


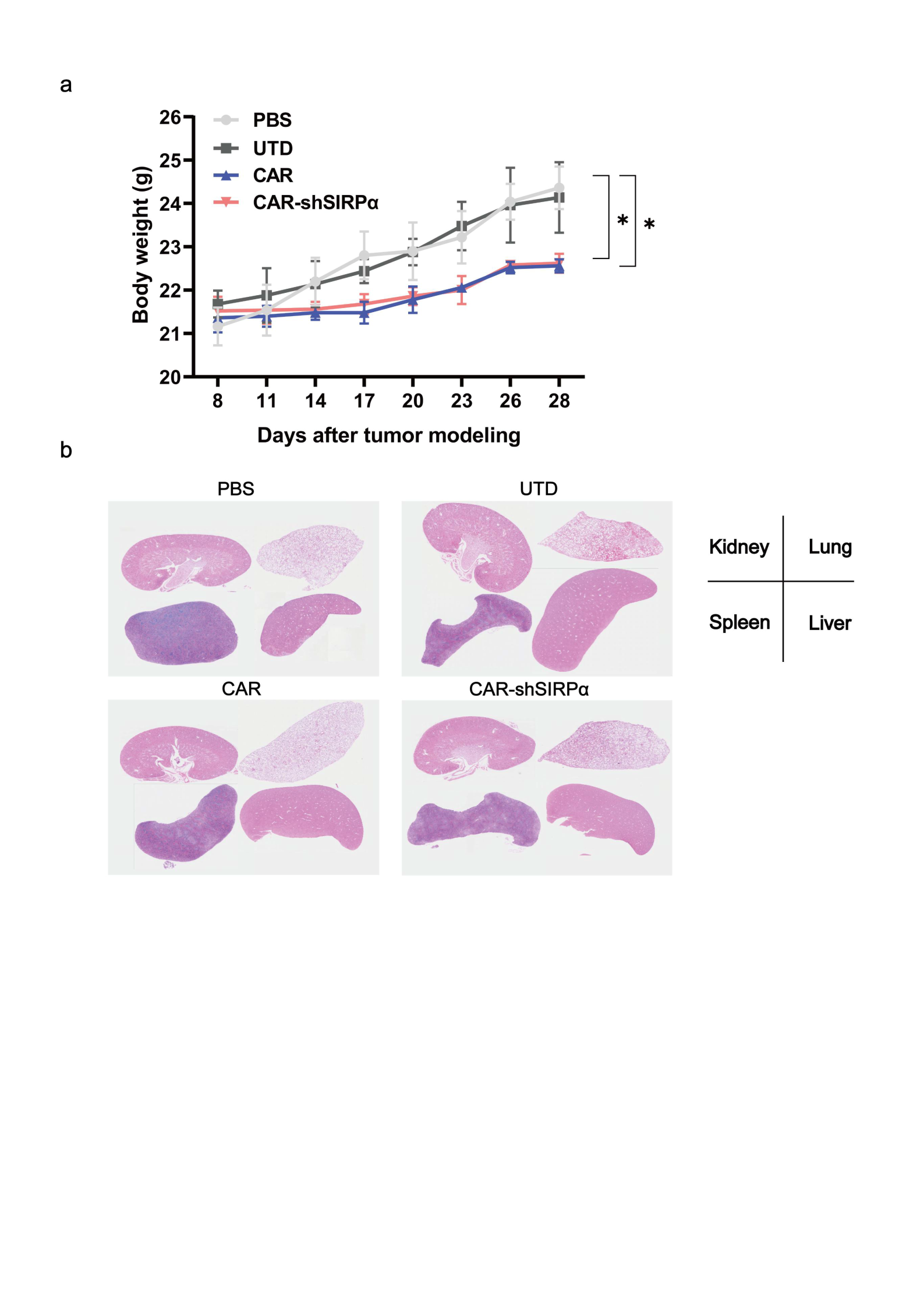


**Supplementary Figure 9: Safety assessment of CAR-modified macrophages for *in vivo* therapy**

**a**, Measurements of body weight in subcutaneous B16-HER2 tumor-bearing mice during CAR-modified macrophage therapy. b, Hematoxylin and eosin (HE) staining of the kidney, lung, spleen, and liver following three weeks of CAR-modified macrophage treatment.


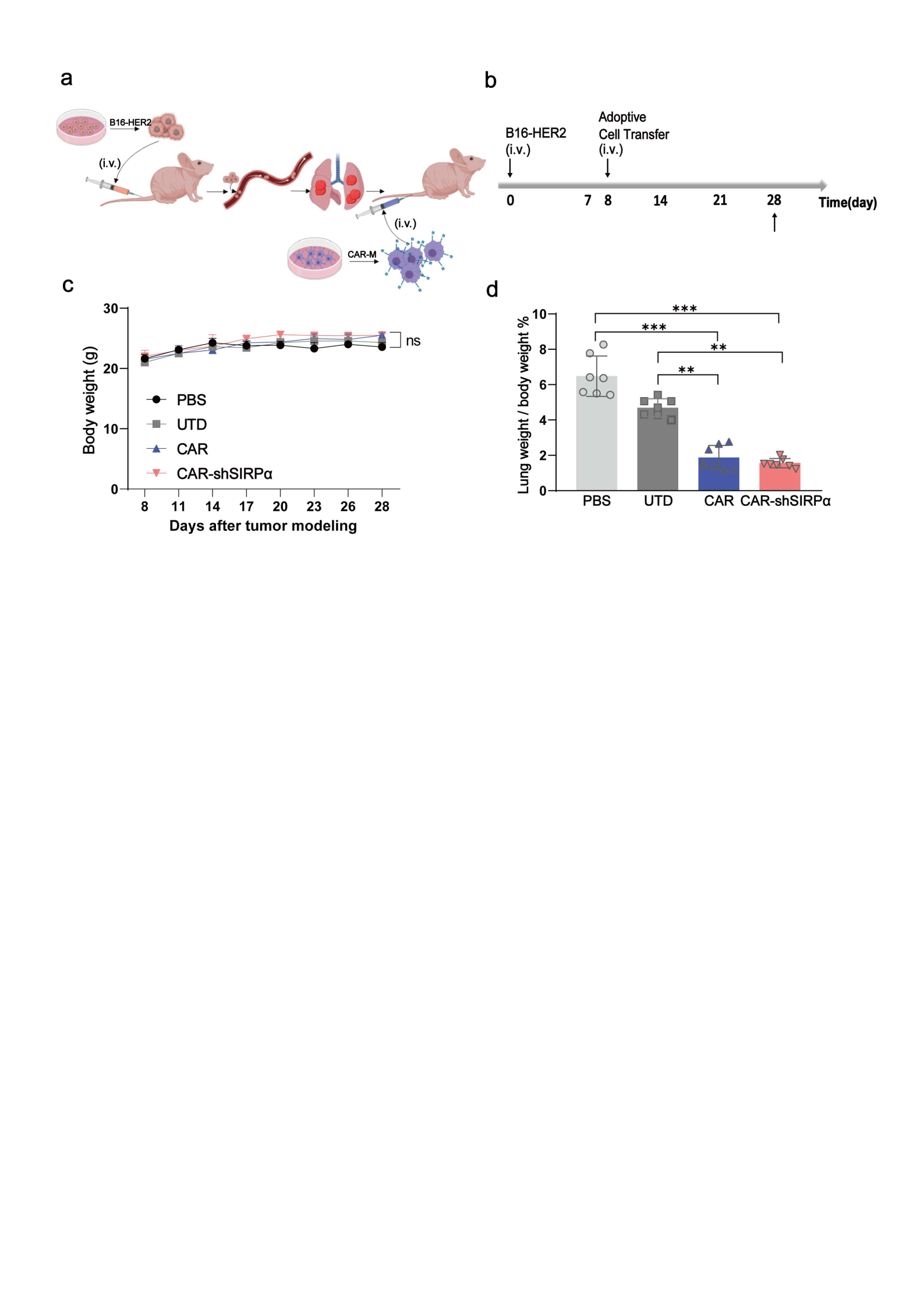


**Supplementary Figure 10: CAR-modified macrophages therapy significantly reduce tumor growth in a lung metastasis model**

**a**,**b**, Establishment of a lung metastatic tumor model through tail vein injection in nude mice. (a) Schematic diagram. (b) Timeline of relevant procedures. **c**, Body weight measurements of tumor-bearing mice during CAR-modified macrophage therapy. **d**, The ratio of lung weight to body weight in tumor-bearing mice during CAR-modified macrophage therapy.


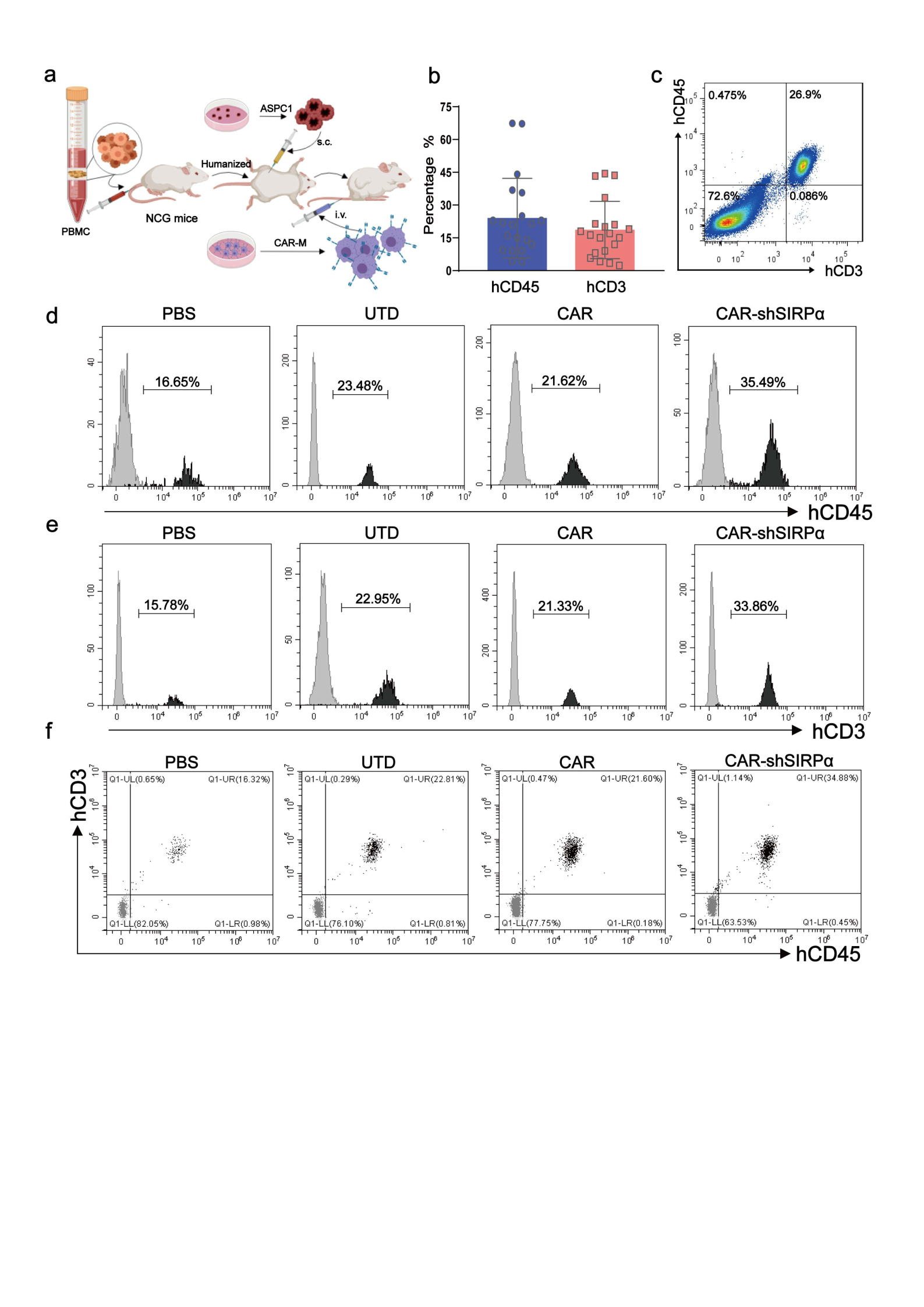


**Supplementary Figure 11: T cell activation in the peripheral blood of CAR-shSIRPα-M treated tumor-bearing humanized immune system (HIS) mice**

**a**, Graphic illustration of the construction and treatment of tumor-bearing HIS mice. **b**, Percentage of human CD45^+^ (hCD45^+^) and CD3^+^ (hCD3^+^) T cells in the peripheral blood of HIS mice two weeks after hu-PBMC injection. Each point represents a single mouse (n = 20 mice per group). **c**, FACS analysis of the percentage of hCD3^+^ T cells among hCD45^+^ cells in the peripheral blood of HIS mice. **d**, **e**, Representative flow cytometry analysis diagrams of hCD45^+^ (d) and hCD3^+^ (e) T cells in the peripheral blood of tumor-bearing HIS mice two weeks after CAR-modified macrophages treatment. **f**, Representative flow cytometry analysis diagram of the percentage of hCD3^+^ T cells in the peripheral blood hCD45^+^ cells of tumor-bearing HIS mice receiving CAR-modified macrophages injection for 2 weeks.


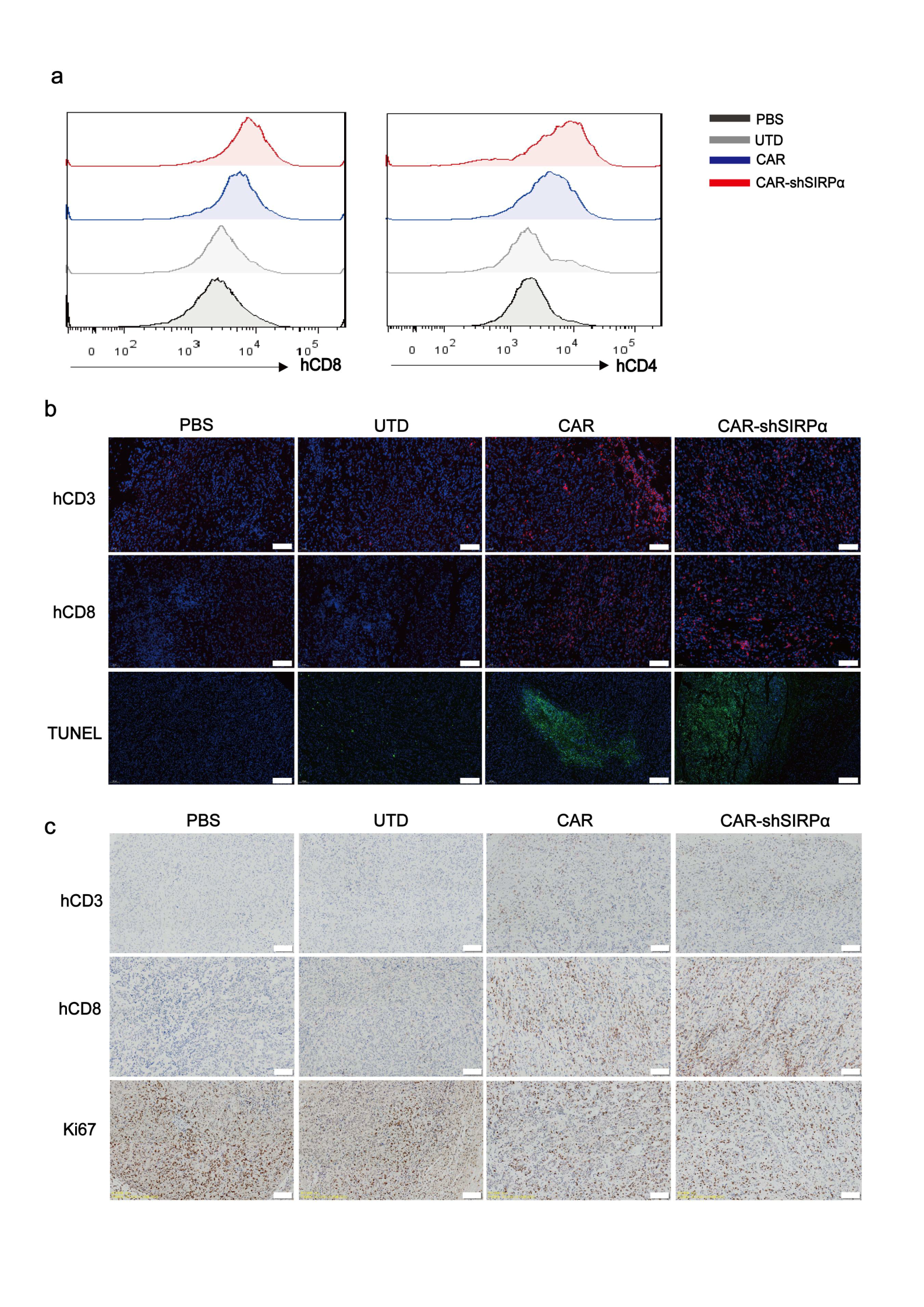


**Supplementary Figure 12: T cell tumor infiltration in tumor-bearing HIS mice**

**a**, Tumors were excised and processed into single-cell suspensions four weeks after implantation in the HIS mice, and the levels of hCD8 and hCD4 expression within the tumors were assessed by flow cytometry. **b**, **c**, Immunohistochemistry (IHC) staining (b) and immunofluorescence (IF) staining (c) of hCD3, hCD8, and Ki67 or TUNEL were analyzed in the tumor sections obtained at the four-week mark after CAR-modified macrophage treatment in tumor-bearing HIS mice.


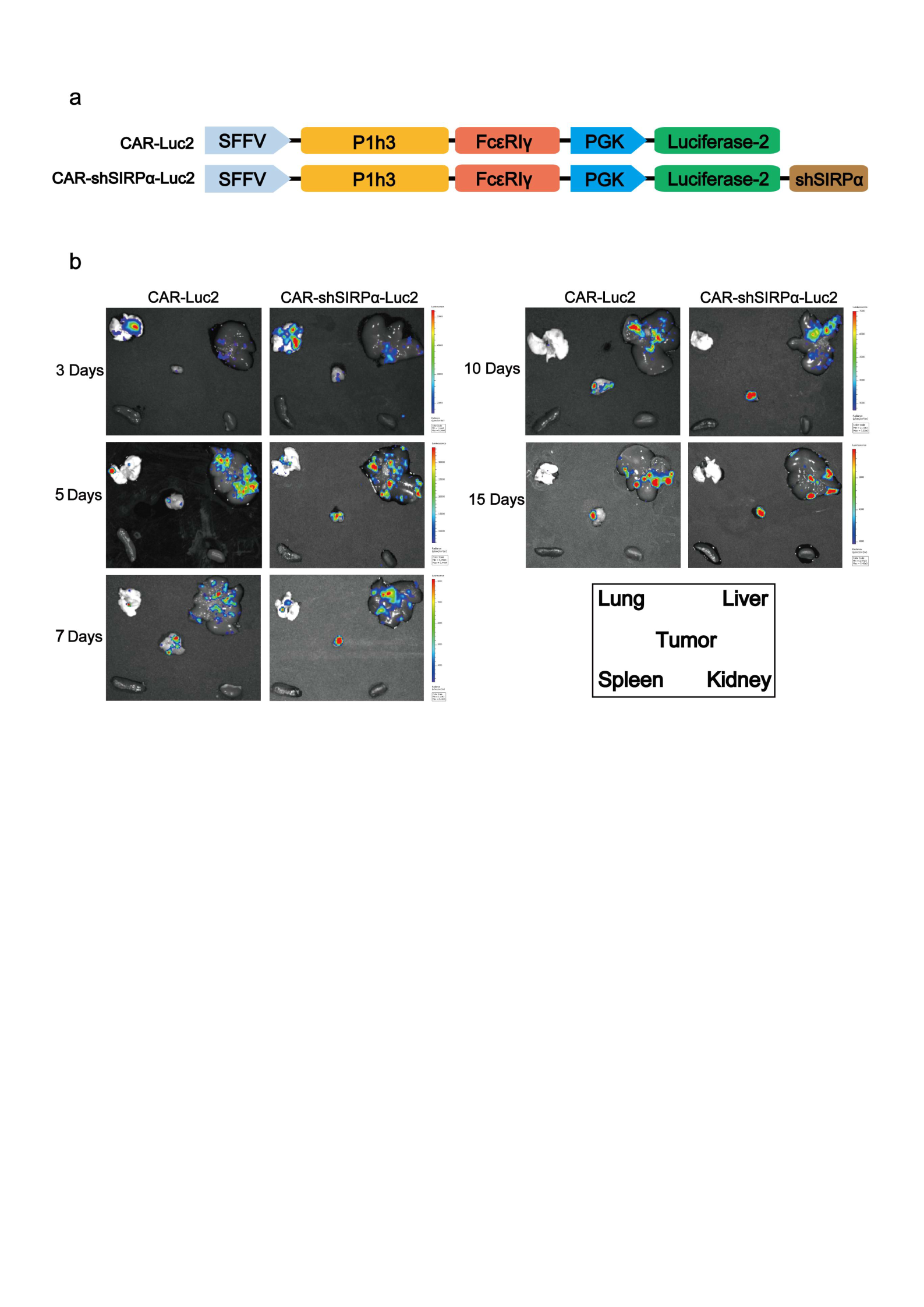


**Supplementary Figure 13. Distribution of CAR-Modified Macrophages in Main Tissues**

**a**, Structural diagram of CAR-luc2 and CAR-shSIRPα-luc2. **b**, B16-HER2 subcutaneous tumor-bearing mice receiving CAR-modified macrophages *via* intravenous administration were sacrificed at each indicated time point. The lung, liver, spleen, kidney, and tumor were isolated, and the distribution of CAR-luc2 and CAR-shSIRPα-luc2 macrophages was detected using BLI.
