# Supplementary material for: Silencing of SIRPα enhances the antitumor efficacy of CAR-M in solid tumors
Source: Cell Mol Immunol. 2024 Oct 8;21(11):1335–49. doi: 10.1038/s41423-024-01220-3 (PMC11527885; doi:10.1038/s41423-024-01220-3)

Source Figure 1g

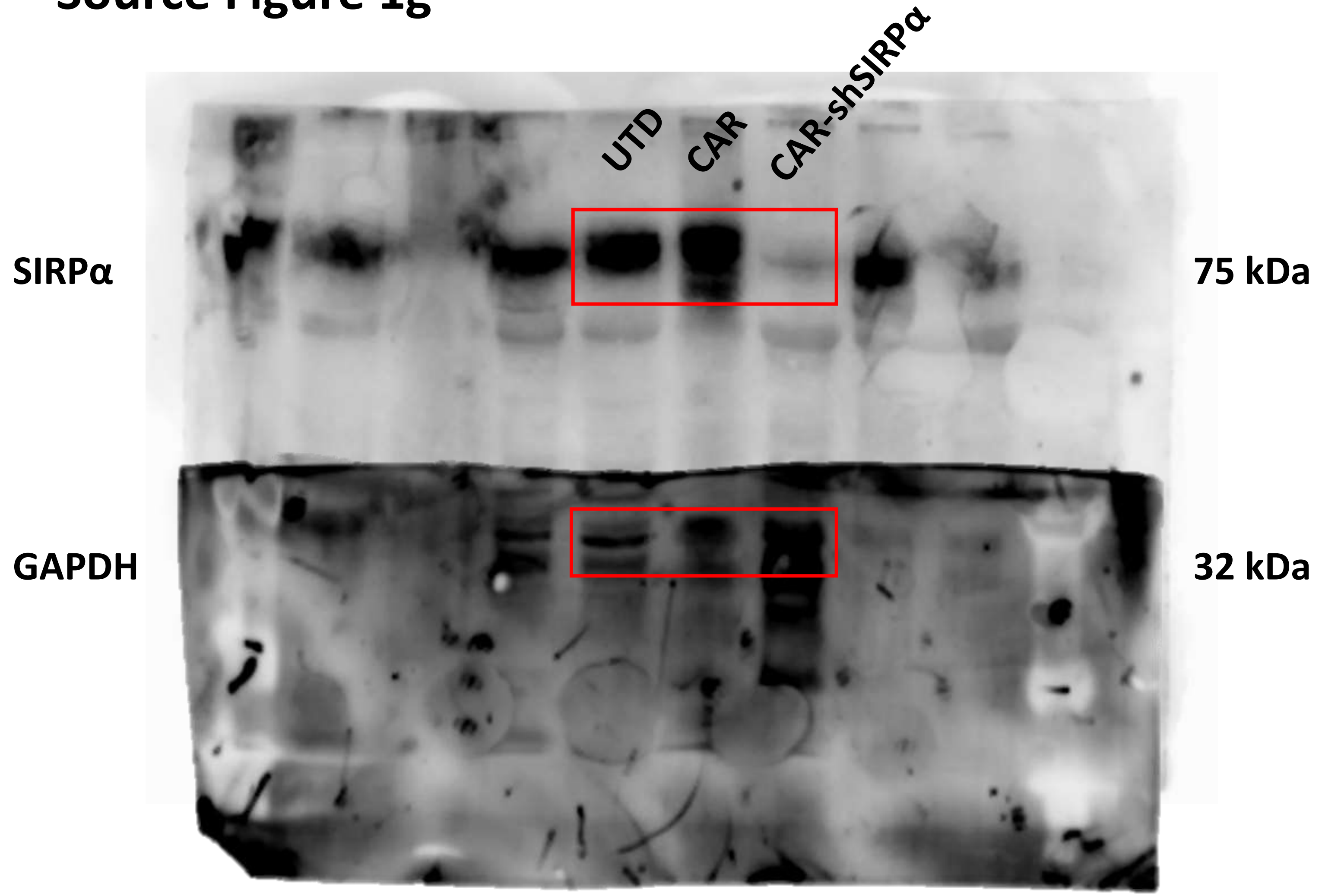

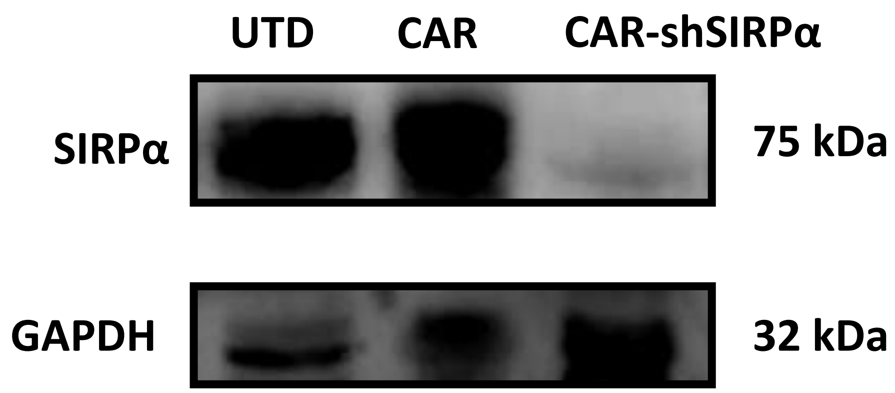

Source Figure 5i

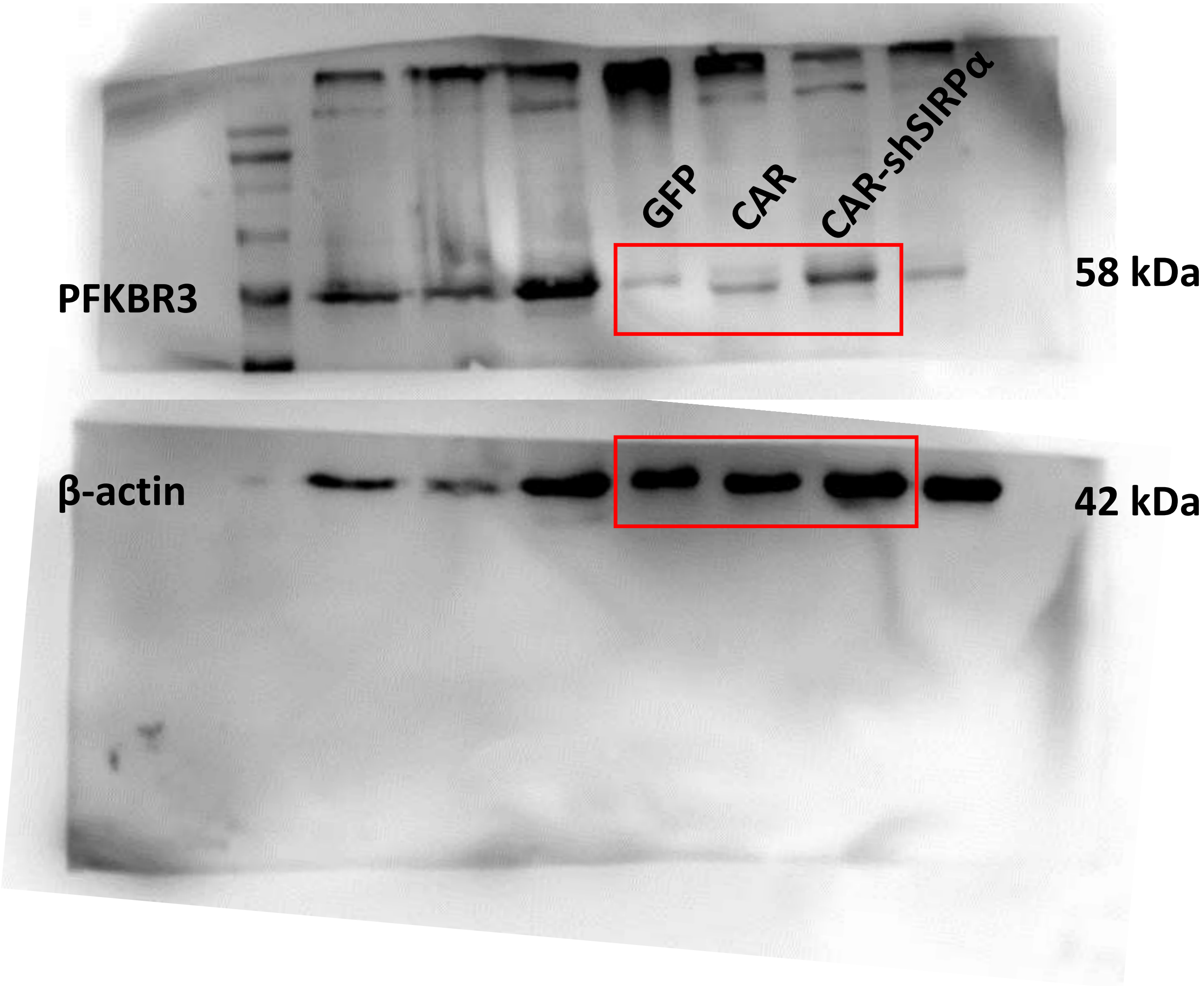

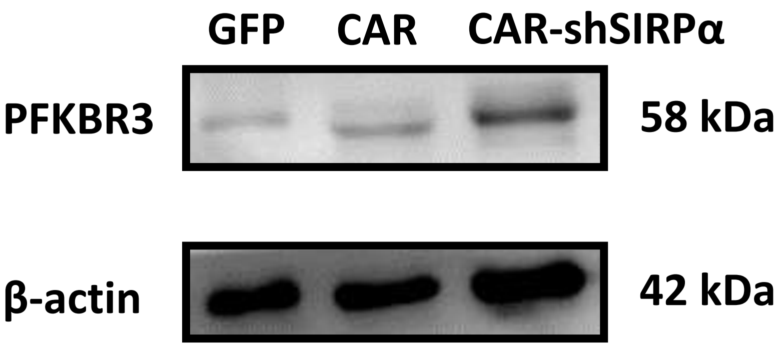

Source Figure 5i

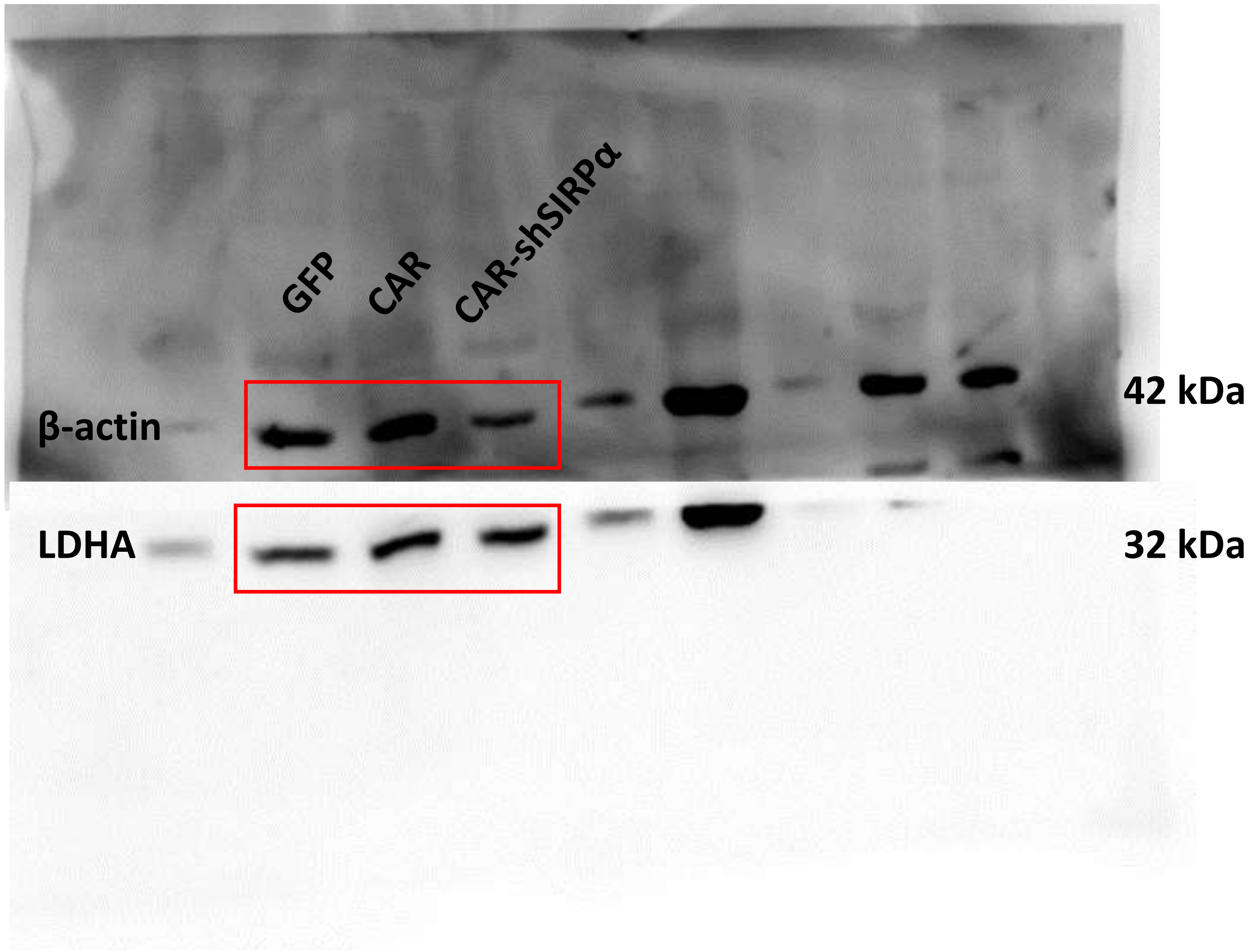

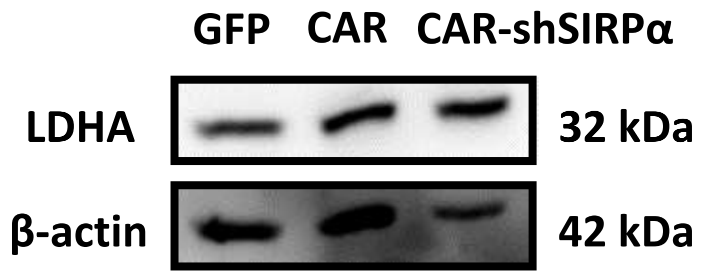

Source Figure 5l

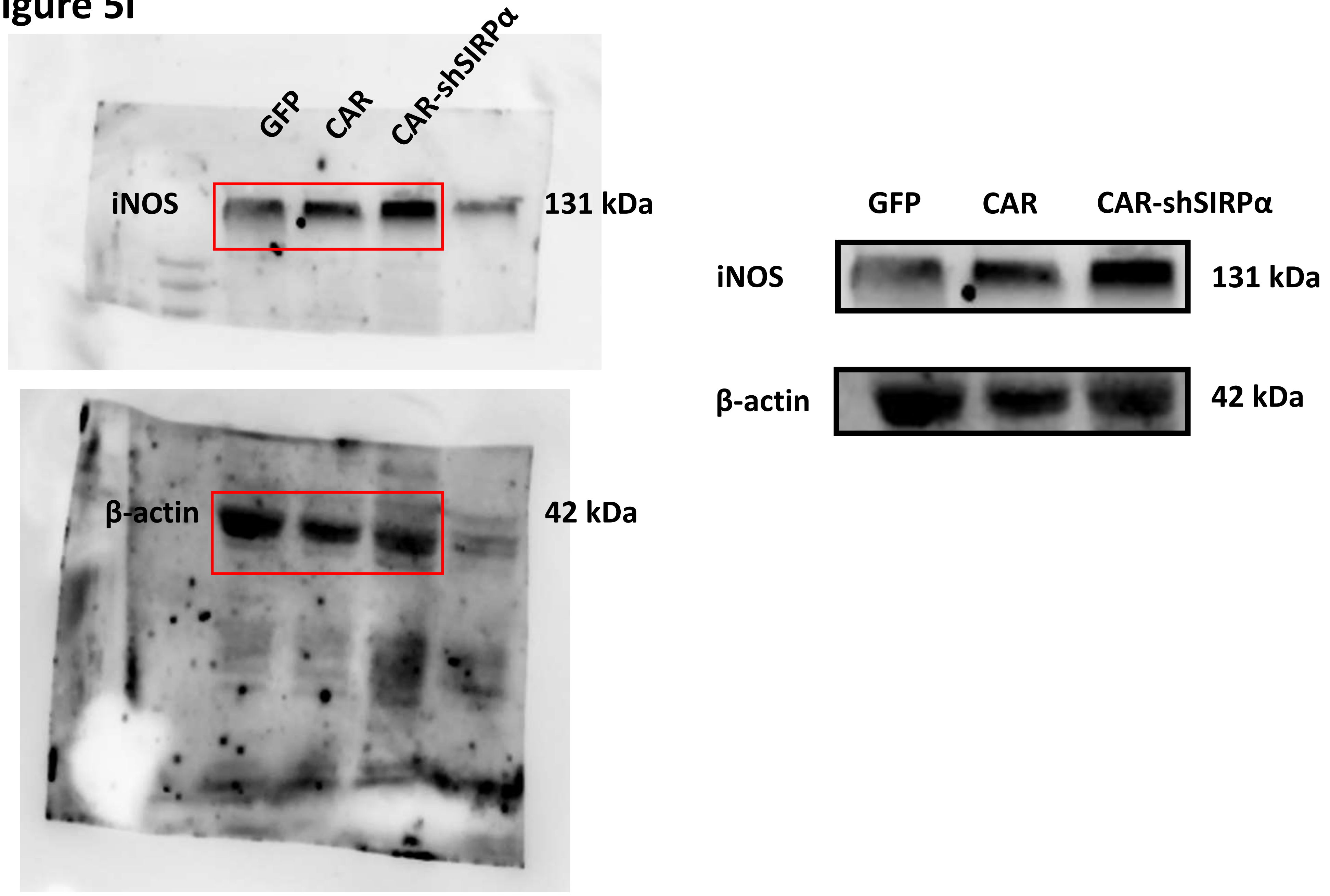

Source Figure 5j

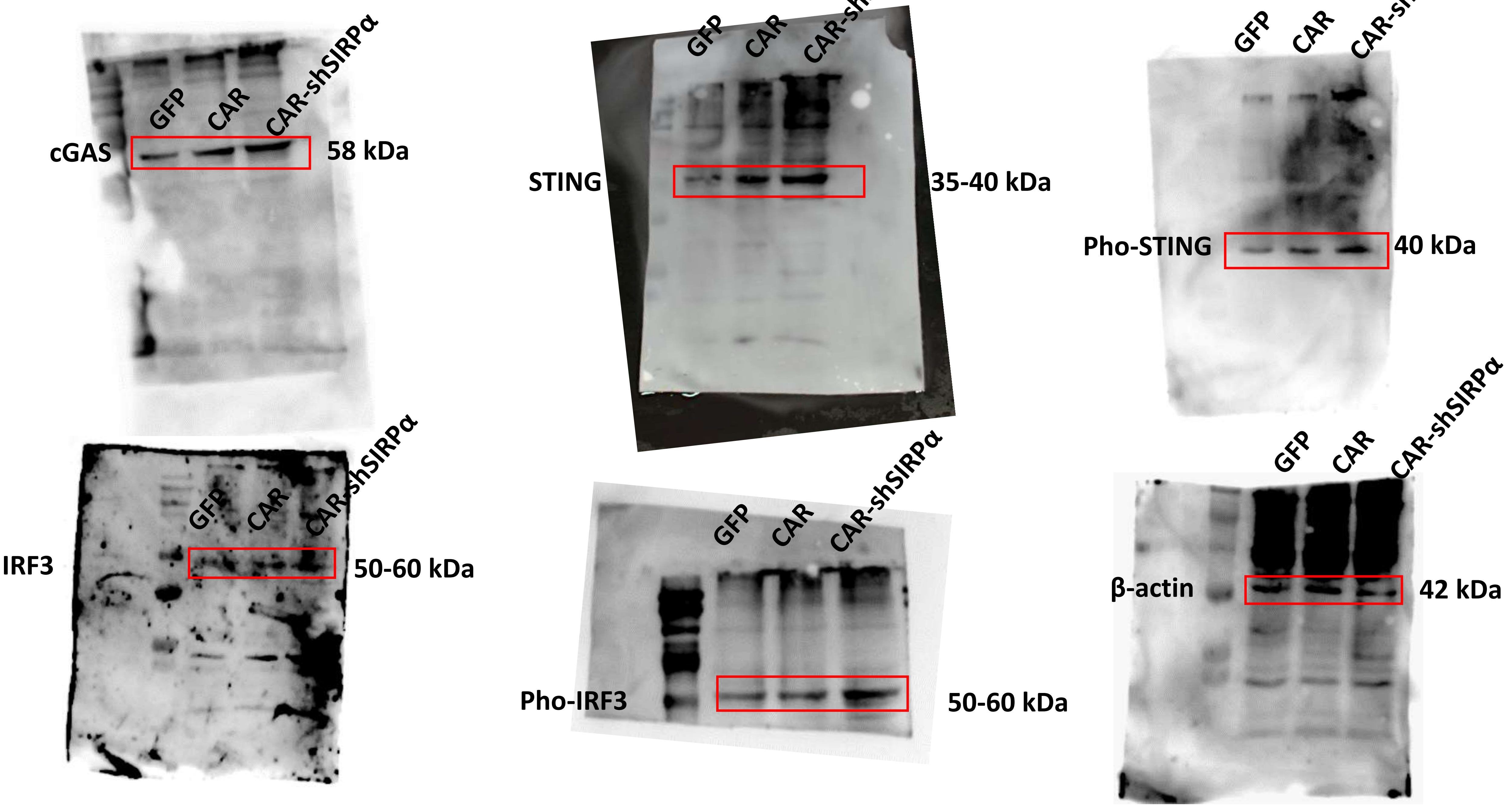

Source supplementary Figure 2e

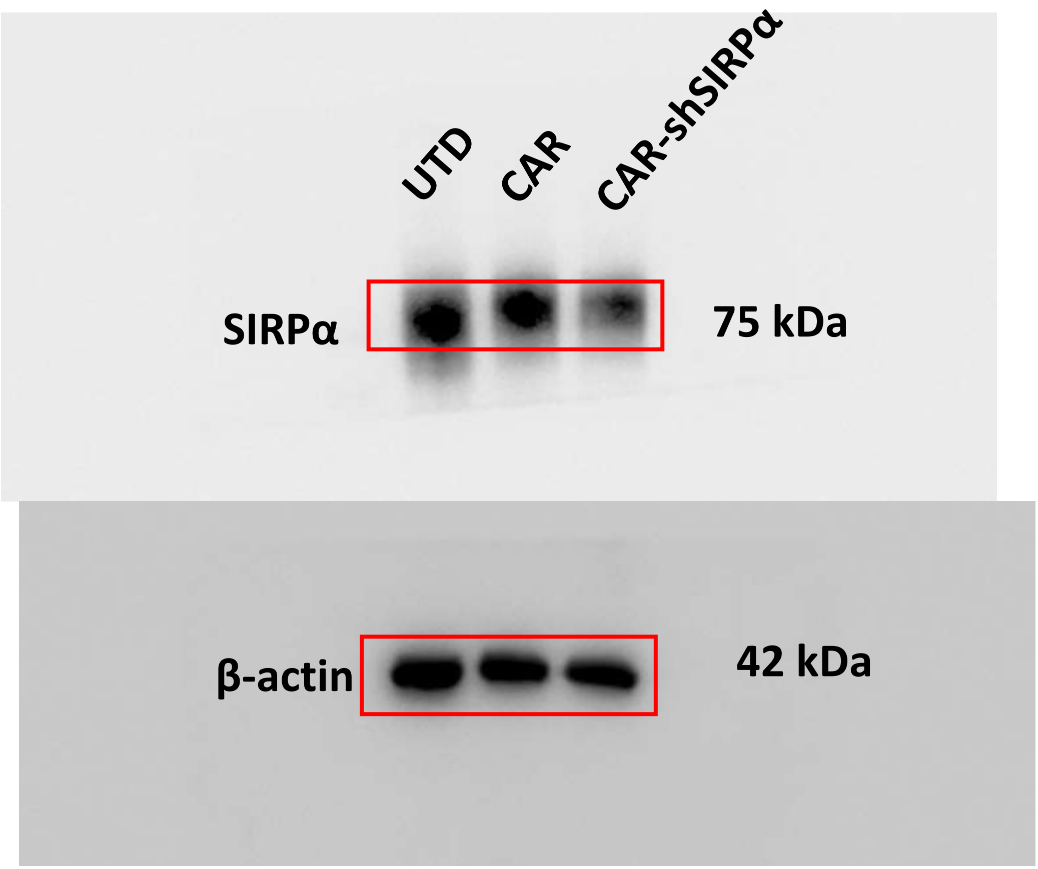

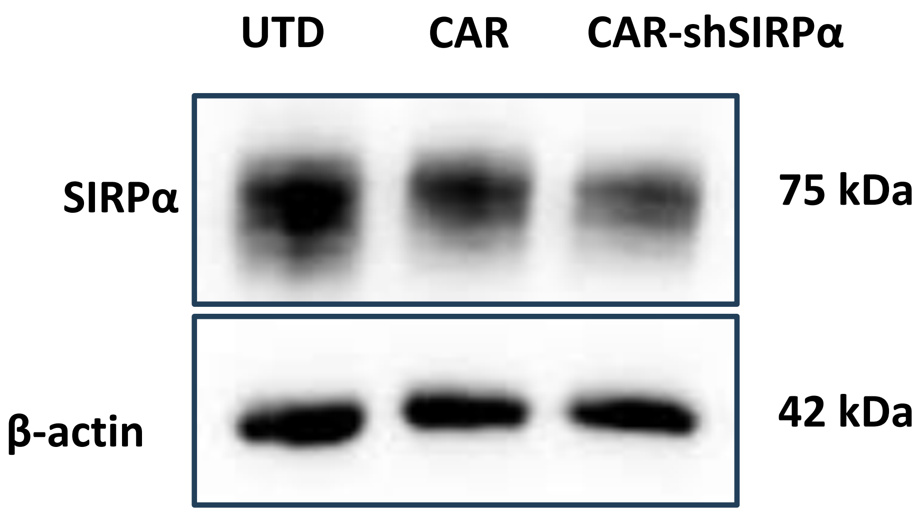

Supplement: Supplementary file 2 — Original image of WB [file 41423_2024_1220_MOESM2_ESM.pdf]
